# An integrated *in silico* approach for functional and structural impact of non- synonymous SNPs in the *MYH1* gene in Jeju Native Pigs

**DOI:** 10.1186/s12863-016-0341-1

**Published:** 2016-02-04

**Authors:** Mrinmoy Ghosh, Simrinder Singh Sodhi, Neelesh Sharma, Raj Kumar Mongre, Nameun Kim, Amit Kumar Singh, Sung Jin Lee, Dae Cheol Kim, Sung Woo Kim, Hak Kyo Lee, Ki-Duk Song, Dong Kee Jeong

**Affiliations:** Department of Animal Biotechnology, Faculty of Biotechnology, Jeju National University, Jeju-Do, 690-756 Republic of Korea; Sher-e-Kashmir University of Agricultural Sciences and Technology, R.S. Pura, Jammu India; Department of Animal Biotechnology, College of Animal Bioscience and Technology, Kangwon National University, Chuncheon, 200-701 Republic of Korea; Livestock Promotion Institute, Jeju Special Self-governing Province, Jeju-Do, 690-756 Republic of Korea; Animal Genetic Resources Station, National Institute of Animal Science, Rural Administration, Namwon, Republic of Korea; Department of Animal Biotechnology, Chonbuk National University, Jeonju, 561-756 Republic of Korea

**Keywords:** *MYH1*, Non-synonymous SNPs, Protein-protein interaction, Homologous modeling, Molecular dynamic simulation

## Abstract

**Background:**

This study was performed to identify the non- synonymous polymorphisms in the myosin heavy chain 1 gene (*MYH1*) association with skeletal muscle development in economically important Jeju Native Pig (JNP) and Berkshire breeds. Herein, we present an *in silico* analysis, with a focus on (a) *in silico* approaches to predict the functional effect of non-synonymous SNP (nsSNP) in *MYH1* on growth, and (b) molecular docking and dynamic simulation of MYH1 to predict the effects of those nsSNP on protein-protein association.

**Results:**

The NextGENe (V 2.3.4.) tool was used to identify the variants in *MYH1* from JNP and Berkshire using RNA seq. Gene ontology analysis of *MYH1* revealed significant association with muscle contraction and muscle organ development. The 95 % confidence intervals clearly indicate that the mRNA expression of *MYH1* is significantly higher in the Berkshire *longissimus dorsi* muscle samples than JNP breed. Concordant *in silico* analysis of MYH1, the open-source software tools identified 4 potential nsSNP (L884T, K972C, N981G, and Q1285C) in JNP and 1 nsSNP (H973G) in Berkshire pigs. Moreover, protein-protein interactions were studied to investigate the effect of MYH1 mutations on association with hub proteins, and MYH1 was found to be closely associated with the protein myosin light chain, phosphorylatable, fast skeletal muscle MYLPF. The results of molecular docking studies on MYH1 (native and 4 mutants) and MYLFP demonstrated that the native complex showed higher electrostatic energy (−466.5 Kcal mol^−1^), van der Walls energy (−87.3 Kcal mol^−1^), and interaction energy (−835.7 Kcal mol^−1^) than the mutant complexes. Furthermore, the molecular dynamic simulation revealed that the native complex yielded a higher root-mean-square deviation (0.2–0.55 nm) and lower root-mean-square fluctuation (approximately 0.08–0.3 nm) as compared to the mutant complexes.

**Conclusions:**

The results suggest that the variants at L884T, K972C, N981G, and Q1285C in MYH1 in JNP might represent a cause for the poor growth performance for this breed. This study is a pioneering in-depth *in silico* analysis of polymorphic *MYH1* and will serve as a valuable resource for further targeted molecular diagnosis and population-based studies conducted for improving the growth performance of JNP.

**Electronic supplementary material:**

The online version of this article (doi:10.1186/s12863-016-0341-1) contains supplementary material, which is available to authorized users.

## Background

*Sus scrofa* was domesticated over 9,000 years ago and has become one of the most important farm animals [1, 2]. The use of porcine offers distinct advantages over the use of other nonrodent animals for studies on physiological, anatomical, pathological, and genomic variations within species, and has also been recommended as a potential model species for investigation of topics related to human health [3, 4]. Therefore, the choice of pig as a non-rodent animal can benefit both livestock and biomedical researches [5].

The functional capacity of skeletal muscle depends on both the quality and the quantity of muscle proteins. Different muscle proteins are synthesized at dissimilar rates [6] and are regulated by distinct genes [7]. Skeletal muscle genes are potential candidate genes that can functionally influence livestock production and meat quality [8]. The diversity in the morphological and biochemical properties of skeletal muscle is unique to this tissue and could arise as a result of the types of protein present, which depends on the genes that are expressed [9]. Research on the relationships between skeletal muscle characteristics and meat quality is crucial for improving our understanding of the molecular basis of skeletal muscle phenotypes [10]. The growth performance of meat animals is related to the composition of the muscle fiber types, and therefore changes in this composition have been proposed to be a modulator of animal growth [11].

Myosin is the most abundant protein expressed in striated muscle cells: myosin makes up ∼ 25 % of the total protein pool and is the main contractile protein that converts chemical energy into mechanical energy through ATP hydrolysis [8, 12]. In mammals, 10 distinct myosin heavy chain (*MYH*) isoforms have been studied. These isoforms are mainly expressed in skeletal muscle during different developmental stages, including the embryonic period, and therefore play a role in the development of skeletal muscle [13]. For instance, *MYH1*, which encodes isoform 1 of *MYH* family, is substantially involved in the metabolism and development of skeletal muscle [14, 15]. The 5,866-bp mRNA of pig *MYH1* gene (Chr. 12:57965087…57984759) encodes with 1,939 residues. *MYH1* is critically important for fast and slow skeletal muscle development, thus it can impact on the overall development [16].

Porcine a key source of meat are widely consumed in several countries. During the last decade, pork meat quality has been targeted in large breeding programs, and has therefore been the focus of a substantial amount of research [14, 17]. In South Korea, Jeju Island represents an exotic natural environment that has its own distinct livestock resources. Jeju Native Pig (JNP), an indigenous breed of swine that is found at Jeju-Do, is particularly desired by consumers because its meat is delicious and is more tender and marbled than the meat of Landrace and Western breeds [18]. However, low feed efficiency, small litter size and small adult body weight are major drawbacks of the JNP breed [19]. By contrast, the Berkshire breed gains weight more efficiently and yields leaner meat as compared to Landrace and Western breeds. Moreover, it’s closely related to Asian native pigs [20]. Consequently, farmers have switched from traditional pig farming to farming specifically for muscular traits, because Berkshire pigs represent a highly favorable choice for farmers who seek to raise livestock that consumers appreciate.

The identification of genes that determine body composition is of major interest in studies aimed at improving livestock production. To date, various RNA-seq approaches have been employed for investigating differential gene expression or for comparing the transcriptomes of distinct muscles in the pig using next-generation sequencing (NGS)-based methods, and has generated new insights into the molecular basis of myogenesis [21, 22]. Comparative transcriptome analysis of muscle tissues revealed differential expression of *MYH1* in JNP and Berkshire breeds. Moreover, *MYH1* was one of 8 myofibrillar genes previously reported to be differentially expressed during prenatal development of skeletal muscle in Berkshire and JNP breeds [21].

The use of NGS methods serves as a powerful approach for generating massive amounts of genome-wide sequence-variation data, and therefore bioinformatics tools are being developed to provide computational predictions on the functional effects of sequence variations [18, 23]. The ultimate goal when using these tools is to identify deleterious non-synonymous SNP (nsSNP) that might lead to alterations in protein structure and thus account for their effect on protein functions. Given the functional importance of nsSNP, our aim here was to identify highly deleterious nsSNP associated with *MYH1* in JNP and Berkshire pigs using various online bioinformatics algorithms.

We conducted the detection approach based on utilizing the RNA-seq gene expression to identify the mutational variants in *MYH1* gene of two breeds. Further, quantitative trait loci (QTL) are biologically meaningful loci at which gene expression is modified by genotype. Accordingly, we utilize the QTL map in SSC12 (*Sus scrofa* chromosome 12) from recently published studies as a means to assess the integrity of sequencing. Currently, a network approach is increasingly used in biological and genetic studies to gain insight into the connections between proteins that collaboratively generate complex traits [24, 25]. The patterns of hub proteins can play central roles in modules [22, 26]. Therefore, we also investigate whether polymorphisms in *MYH1*affect the interaction with hub proteins. The steps we present herein ultimately provide a straightforward approach that allows for more accurate identification of specific variations in the JNP and Berkshire breeds responsible for quantitative traits that can be confidently used as selection markers for improving growth performance.

## Results

### Functional annotation of *MYH1*

Transcriptome data were acquired from muscle tissue samples of JNP and Berkshire using Ilumina HiSeq2000. The generated data consisted of highly reliable RNA-seq reads on an average 90.8 % (muscle) reads passed the quality control. These sequences were mapped successfully to the *S. scrofa* genome using TopHat (v2.0.2). The significant difference (*P* < 0.01) expression of genes identified by the general linear model.

The detailed of function ontology, revelation of common processes and the pathways potentially associated with *MYH1* were investigation. The GO analysis related to the biological process of the different identified gene was examined (Additional file 1: Table S1). Gene ontology (GO) revealed the significantly association of *MYH1* with the muscle contraction (GO: 0006936) and muscle organ development (GO: 0007517) categorized under the biological process (Additional file 1: Figure S1). The process of muscle organ development is also related to certain putative *MYH1* functions. The biological pathway analysis suggested that the candidate gene was associated with 6 pathways related to metabolic processes, inflammatory response, and control of muscle growth (Additional file 1: Table S2). The *P*-values (significance) obtained for *MYH1* pathways are shown in Additional file 1: Figure S2; these values varied from 0.005 to 0.025, and maximal significance (*P* = 0.005) was for the pathway “Translocation of GLUT4 to the Plasma Membrane.”

### Validation of *MYH1* mRNA expression in muscle tissues of JNP and Berkshire breeds

**Fig. 1:**
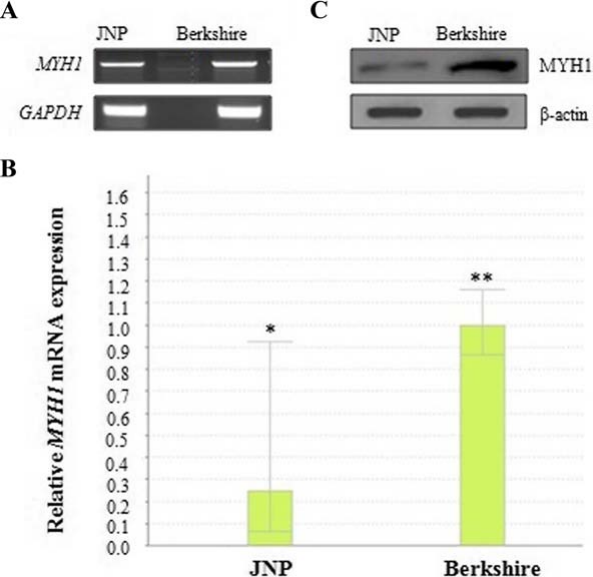
Expression analysis of mRNA of *MYH1* in JNP and Berkshire. **a** Expression of *MYH1* mRNA in 1 % agarose gel at RT-PCR. **b** Relative quantitative has shown the significant expression differences of *MYH1* gene between two breeds. Error bars represent the 95 % confidence interval. **c** Relative differential blot expression analysis of MYH1 proteins in JNP and Berkshire

Both RT-PCR and real-time qRT-PCR were performed to clarify the qualitative and quantitative expressions of the gene under study. Specifically, the relative mRNA expression levels of *MYH1* were determined by normalizing the levels of the 5 pigs from each JNP and Berkshire breeds against the transcript levels of an endogenous reference gene, *GAPDH*. The 95 % confidence intervals clearly indicate that the mRNA expression of *MYH1* is significantly higher in the Berkshire *longissimus dorsi* muscle samples than in JNP pig (Fig. 1a and b). The results obtained for the differential transcript levels from real-time PCR analysis complemented the findings obtained by RNA-Seq. In addition, the protein expression for the MYH1 obtained by western blot has also been presented as relative band intensities between the breeds (Fig. 1c). MYH1 protein was expressed at a significantly (*P* < 0.05) higher level in Berkshire against the counterpart.

### Annotation of QTLs at *MYH1* region and linkage disequilibrium

The scatter plot was constructed on the basis of expression levels of selected genes and the data are visualized on a log scale. The dotted purple line in the scatter plot reflects the linear regression (R^2^)/‘best-fit’ line are crosses close to as many data points as possible. The analysis of relative expression showed that copy number ration (CNR) in Berkshire was found as 8.75 log_2_ whereas 8.97 log_2_ in JNP (Additional file 1: Figure S3 A).

Further the QTL analysis based on the previously reported documentation on *MYH1*region at chromosome 12 has shown total number of 8 QTLs significantly associated with growth quantitative traits (Additional file 1: Figure S3 B). In details, two QTLs (QTL ids 5717 and 5949) were associated with body weight, whereas the association of single QTL for ham hat thickness (QTL id 3966), back fat thickness (QTL id 5990), meat quality and marbling (QTL id 3288) were identified (Table 1).

**Table 1:** Summary of the QTLs in porcine *MYH1* at SSC12 associated with meat quality traits

QTL ids	Peak cM	Marker	*P* value (<0.05)	Identified QTLs	Reference (Pubmed id)
5717	93.5	SW874, SW605	Significant	BW	17459017, 24797173
5949	97.4	SW874, SW605	Significant	BW	18712441
3966	69.3	SW605	Significant	HFT	17121599
5990	101.2	SW874, SW605	Significant	BFT	18712441
3288	84.8	SW2180, S0090	Significant	MQ; MARB	17965326; 25678226, 12081803, 18239890
12844	106.6	S0106, SWR1021	Significant	ADIPDI	20667088
3824	107.8	SWC23, SW2180	Significant	10RIBBFT	15318717
21403	N/A	SWC23, SW2180	Significant	CIE a	22303314

*BW* Body Weight, *HFT* Ham Fat Thickness, *BFT* Back Fat Thickness, *MQ* Meat Quality, *MARB* Marbling, *ADIPDI* Adipocyte Diameter, *10RIBBFT* Backfat at Tenth Rib

**Fig. 2:**
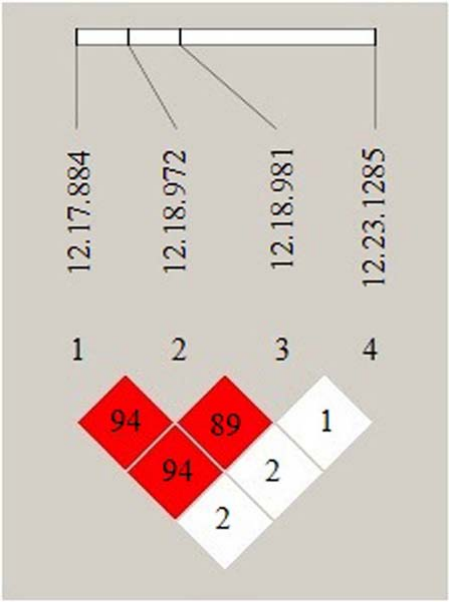
Haploview plot of linkage disequilibrium (r^2^) between significant and suggestive SNP on chromosome. The numbers from 1to 4 represent the selected SNP. The diamond shaped with a number represents linkage disequilibrium of SNP

Sequencing of *MYH1* revealed all the 10 SNPs in JNP and 3 SNPs in Berkshire were situated in exon regions of the gene. The regions on chromosome 12 at CDS 17 and 18 of *S scrofa* were significant associated with metabolism and development of muscle. For further genetyping investigation, we selected K972C, N981G, L884T, Q1285C in JNP. The results obtained from haploview showed that SNP in this region were at strong linkage disequilibrium with each other (r^2^ ranging from 0.84 to 0.94) (Fig. 2). The disequilibrium evidence of K972C (12.18. 972) and N981G (12.18. 981) have shown the strong linkage with L884T (12.17. 884), rather than the Q1285C (12.23.1285).

### Integrate the polymorphisms in JNP and Berkshire breeds

Data on *MYH1* polymorphisms from the 2 breeds were retrieved using NextGENe software. These polymorphisms included 12 downstream, 3 upstream, 26 synonymous, 5 splice-site variants, and 10 missense variants in the JNP breed; whereas 18 downstream, 6 upstream, 8 splice-site variants, 20 synonymous and 3 missense variants are identified in Berkshire breed. To determine whether the identified missense mutations affected *MYH1* function, we further analyzed the missense variants from the JNP and Berkshire breeds using *in silico* prediction algorithms. Our association analysis has shown the skewed distribution of these nsSNP in Jeju Native and Berkshire breeds.

### Effect of predicted deleterious nsSNP changes on residual

Various computational tools were used to estimate the possible effects of the stabilizing residues on protein functions. We applied 6 *in silico* SNP prediction algorithms: SIFT, PROVEAN, PolyPhen-2, I-Mutant 3.0, PANTHER, and Project HOPE respectively. The details of the scores of the nsSNP analysis are summarized in Table 2.

**Table 2:** The non-synonymous single amino acid variation in *MYH1* from Jeju Native Pig and Berkshire

Chr. position	CDS	Position and substitutions of amino acid	SIFT	PROVEAN (cutoff = −2.5)	PolyPhen2	I-Mutant 3.0 (DDG^a^Kcal/mole)	PANTHER^b^	
							subPSEC	P_deleterious_
JNP								
12.57978043	17	M881R	0.00	−3.116	0.544	−0.62	−2.39	0.35
12.57978052	17	L884T	0.00	−3.652	0.964	−1.92	−4.25	0.77
12.57978091	17	A897T	0.03	−2.363	0.001	−0.66	−2.03	0.27
12.57979374	18	K970A	0.00	−5.027	0.913	−0.25	−2.04	0.27
12.57979379	18	K972C	0.00	−6.630	1.000	−0.31	−5.38	0.91
12.57979382	18	H973G	0.00	−7.257	0.443	−0.44	−2.55	0.38
12.57979388	18	T975V	0.00	−3.986	0.023	−0.29	−2.64	0.41
12.57979406	18	N981G	0.00	−4.794	0.053	−0.61	−3.08	0.52
12.57981606	23	Q1285C	0.00	−4.127	0.889	−0.82	−3.88	0.70
12.57981608	23	T1286P	0.01	−3.669	0.976	−0.36	−2.92	0.48
Berkshire								
12.57978091	17	A897T	0.03	−2.207	0.724	−0.66	−2.03	0.27
12.57979342	18	H973G	0.00	−7.257	1.000	−0.44	−2.55	0.38
12.57981608	23	T1286P	0.01	−3.669	0.976	−0.36	−2.92	0.48

^a^DDG < −0.5: Large Decrease of Stability; DDG > 0.5: Large Increase of Stability

^b^A cutoff of −3 corresponds to a 50 % probability that a score is deleterious

According to the SIFT results, all missense variants were categorized as deleterious in both breeds. To predict any deleterious effects, missense were further submitted in PROVEAN to determine deleterious score. The total number of 7 missense variants from JNP such as L884T, K970A, K972C, H973G, N981G, Q1285C, and T1286P were identified as deleterious SNP; conversely, in Berkshire pigs, a deleterious score was found in residual change at H973G.

Given the threshold of the native Bayes probabilistic score, PolyPhen-2 calculates the true-positive rate as a fraction for the predicted mutations. According to our PolyPhen-2 study, 3 amino acid variants (L884T, K972C, and Q1285C) in JNP and 1 variant (H973G) in Berkshire pigs are likely to exert deleterious functional effects on MYH1. In addition, we performed I-Mutant3 analysis for MYH1 in order to add another layer of refinement to the nsSNP characterization. The stability effects were evaluated by subtracting the unfolded Gibbs free-energy value of the native protein from that of the mutated protein. The I-Mutant 3.0 DDG simulation yielded the highest score (−1.92) for L884T in JNP.

The subPSEC score estimates the likelihood of a functional effect arising from a single amino acid substitution using HMM-based statistical modeling and evolutionary relationships. Four amino acid variants (L884T, K972C, N981G, and Q1285C) were found to be deleterious in JNP, with the subPSEC scores ranging from −3 to −5, whereas in Berkshire pigs, all subPSEC scores were < −3 and were therefore classified as tolerant.

MutPred was used to analyze the top-5 functional disruptions caused by the missense mutations. Four amino acid substitutions in JNP—L884T, K972C, N981G, and Q1285C—led mainly to the loss of stability, ubiquitination, phosphorylation, and gain of glycosylation. However, the effect of the mutation in Berkshire was predicted negligible by MutPred (Additional file 1: Table S3). The most common effects were suggested as loss of ubiquitination (*P* = 0.1223), loss of methylation (*P* = 0.1405), gain of phosphorylation (*P* = 0.2086), gain of glycosylation (*P* = 0.2696), and loss of solvent accessibility (*P* = 0.3103).

In each algorithm, distinct parameters are used for evaluating the nsSNP. The higher the number of more positive results obtained for the nsSNP, the more likely they are to be genuinely deleterious. Thus, we selected 4 amino acid variants (L884T, K972C, N981G, and Q1285C) in JNP and H973G in Berkshire pigs for subsequent HOPE analysis.

### Assessment of effects of mutations on protein functions using HOPE

We have selected 4 missense mutations in JNP: L884T, K972C, N981G, and Q1285C for additional study. The original wild-type residue and newly introduced mutant residues often differ in their properties. The PDBePISA (Proteins, Interfaces, Structures and Assemblies) (http://www.ebi.ac.uk/pdbe/pisa/) database contain protein assemblies that are highly likely to be biologically relevant. According to the PISA database, the original residue at the L884T mutation site is involved in multimeric contacts. The mutation introduces a residue at this position that is smaller than the original residue and might not be adequately large for making multimeric contacts. The mutated residue K972C introduces a residue that is less hydrophilic than the original, and that might affect hydrogen-bond formation. The 3D structure of N981G reveals that the wild-type residue is located at α-helix; after mutation this α-helix formation is affected. The conservation scores suggest that these mutations are probably damaging to the protein. In the case of Q1285C, the mutant residue is smaller than the wild-type residue, and this could potentially lead to a loss of external interactions. Moreover, the difference in the hydrophobicity of the wild-type and mutant residues could cause a loss of hydrophobic interactions with other molecules on the surface of the protein.

The wild-type and mutant amino acid (H973G) of MYH1 protein of Berkshire differ in size. The mutant residue is smaller than the wild-type one, which leads to empty space in the core of the protein. In addition, Glycine is extremely flexible and can disrupt the structural rigidity of the protein required at this position. The mutated residue is located on the surface of a domain whose function is still unknown. Nevertheless, the residue at this position could still be in contact with other molecules or domains and such interactions might be affected due to the mutations.

### Prediction the association partners of MYH1

The STRING tool was used to predict the pattern of association (physical and functional) of MYH1 protein with the partner proteins MYLPF, MYL6, ACTG1, RHOA, CGN, TNNI3, TNNC2, MYL1, TNNI2, and TTN. Based on the confidence scores of the MYH1 protein interactions, MYLPF (myosin light chain, phosphorylatable, fast skeletal muscle) was chosen for molecular docking analysis performed with L884T, K972C, N981G, and Q1285C variants of MYH1 (Additional file 1: Table S4).

### Annotation of homology models

The 3D protein structure of *S. scrofa* MYH1 was modeled using Protein Data Bank archive (PDB)-1I84 (Chain S) information, at a resolution of 24.86 Å (Additional file 1: Figure S4). The constructed model was computationally validated using PROCHECK. The darkest areas in Ramachandran plot correspond to the core regions representing the most favorable combinations of Psi/Phi values. All models were scored using discrete optimized protein energy (DOPE), and the model with the lowest DOPE score was considered to be the final model. Overall, 91.1 % of the residues occurred in the most favored region, 7.0 % in additional allowed regions, and 1.1 % in generously allowed regions; only 0.7 % of the residues were in disallowed regions (Additional file 1: Figure S5). The homology models of the 4 mutants (L884T, K972C, N981G, and Q1285C) of MYH1 were also constructed.

Unfortunately, no official model of porcine MYLPF exists in PDB. In this situation, the structure was modeled using the *S. scrofa* MYLPF protein sequence available in the NCBI database. The 3D structures of *S. scrofa* MYLPF were constructed using PDB 2W4A (Chain B) as reference, at are solution of 35.00 Å (Additional file 1: Figure S6). The structures of the modeled proteins show that nearly 85.6 % of the residues occupied favored regions, whereas 10.6 % of the residues occupied the additional allowed regions in Ramachandran plots (Additional file 1: Figure S7). Only 2.3 % and 1.5 % of the residues were present in generously allowed and disallowed regions, respectively.

### Molecular docking of MYH1 (native and mutant) with MYLPF

We studied the molecular docking between MYH1 (native and mutant) and MYLPF in order to identify the variation in the overall interaction energy of the complexes. In the native complex, the electrostatic, van der Waals, and interaction energies were observed to be −466.5, −87.3, and −835.7 Kcal mol^−1^, respectively; by comparison, the corresponding energies for the mutant complexes (L884T, K972C, N981G, and Q1285C) were lower (Table 3).

**Table 3:** Interaction and energy score of native and mutant MYH1- MYLPF complexes

MYH1	Electrostatic energy (Kcal mol^−1^)	van der Walls energy (Kcal mol^−1^)	Interaction energy (Kcal mol^−1^)
Native	−466.5	−87.3	−835.7
L884T	−438.6	−63.2	−784.2
K972C	−428.5	−47.5	−759.4
N981G	−401.0	−32.6	−706.6
Q1285C	−416.3	−41.8	−748.3

### Molecular dynamic simulation to determine the structural stability of native and mutant MYH1-MYLPFcomplexes

**Fig. 3:**
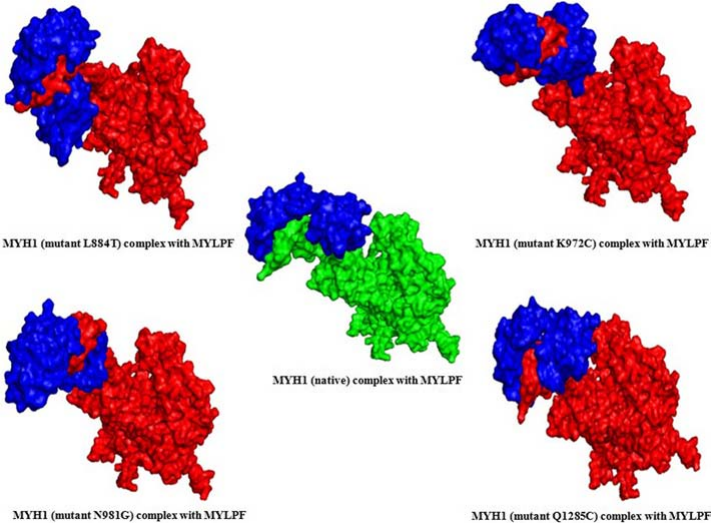
The MYH1 and MYLPF complexes of native and mutants. The molecular dynamic simulation showed the surface structure the conformation changes in MYH1 and four different mutants MYH1-MYLPF complexes. The native MYH1 represented with green surface color and mutant in red whereas MYLPF showed in blue color

Molecular dynamic simulations were performed using 25-ns trajectories. The changes in protein stability were calculated using backbone RMSDs for the native and mutant complexes of MYH1-MYLPF (Additional file 1: Figure S8), and this helped determine the structural deviation of the mutant complexes in comparison with the native complex. We found that the native and mutant complexes deviated in the ranges of 0.2–0.55 and 0.2–0.45 nm, respectively, in simulation times of 5–25 ns. Maximal deviation between the mutant and native complexes occurred at a simulation period of 5–15 ns. The structural flexibility of the complexes was calculated using RMSFs obtained with the 25-ns simulation trajectory values. The fluctuation of native MYH1-MYLFP complex ranged from ~0.08 to ~0.3 nm, and compared with the native complex, the mutant MYH1-MYLFP complexes showed overall higher fluctuation (19–45 nm) (Fig. 3). The changes in the flexibility of the mutant complexes reflect the impact of the nsSNP on MYH1 protein.

## Discussion

Developments in RNA-seq technology dramatic decrease the costs of next-generation sequencing that enable more comprehensive investigation of the transcriptome than microarrays have led to numerous RNA-seq studies in recent years. Despite the increasing economic importance of meat quality, the current research focused on the skeletal muscle transcriptome analysis using cDNA libraries and comparing the gene expression profile in the muscles of different quality.

Present study included a total numbers of 10 animals (*n* = 5 from each breed). The *longissimus dorsi* muscle samples were collected from 8 months full growth adult pigs, considered as the economical tread time period in South Korea when the farmer sold out the livestock for slaughter. The numbers for the experiment animals were decided based on the minimum average relatedness and according to method described by Erb [27] and Charan [28]. Although it was investigated which tissues express *MYH1* and what the effects of sequence polymorphisms are, little is known in the JNP pig about its relative mRNA expression when comparing tissues with the western breed. This knowledge is essential in researching differences in *MYH1* regulation in view of selection for improved meat quality.

The QTL analysis and expression profiling in different porcine resource population are aiming to identify genes controlling meat quality and carcass traits. Porcine *MYH1* serve as a positional and functional candidate gene for meat production in domestic animals. QTL mapping has become common in livestock research have been compiled in the AnimalQTLdb database. The major purpose of QTL mapping is to identify genes or mutations causing variation in quantitative traits. Herein we have found 8 QTLs in *MYH1* at chromosome 12 are associated with body weight, meat quality, marbling, ham and back fat thickness. The various studies on association of genetic loci mapping for meat quality identified novel QTLs of in porcine *MYH* gene family (*MYH1, MYH2, MYH3*, and *MYH13*) which provide new insights into the genetic basis of meat quality trait in pigs [29]. To substantiate the presence of QTL influencing growth associated phenotypes in *Sus scrofa,* our analysis demonstrated that the *MYH1* in 12 chromosome use of aggregation study for meat quality and body growth. The early finding was also reported that QTL on *Sus scrofa* chromosome 12 influencing the growth related traits [30]. In fact it might be consider to one of the principal region on chromosome 12 of the variability of each phenotype of JNP and it is directly related to any of the original biological traits. However, genome-wide analyses on a larger population scale are required for further validation.

The pioneer studies showed the selection based on minimum average relatedness, contributes to high percentage of polymorphic markers and are also indicative signature for homozygosity for each breed [31, 32]. Therefore the less number of animals and throughput analysis can also be representative to state the specificity of the SNP in a breed for the population. Haplotypes have been assigned to each subject by computing the normalized probabilities of the linkage phases of each phenotype [33]. Haplotypes were shown the influence the studied phenotypes. The significant difference in *S* score observed at 12.17.1285 with other three haplotypes. However, a larger population study is still needed to precisely estimate the effects and gene actions of these chromosomal regions on growth trials improvement program.

Previously, defects in the circadian expression of muscle genes were reported to cause large disruptions in the normal expression of genes crucial for adult muscle structure and metabolism [16, 34]. Therefore, we used sequence- and structure-based approaches to investigate the most salient polymorphisms in *MYH1* in JNP and Berkshire breeds. Such missense variants are considered to be potentially critical for the function and structure of a protein, and thus they might provide markers for the selection of specific phenotypic traits [35].

This study is the first attempt to screen residue variants in *MYH1* from JNP and Berkshire pigs using NextGENe. This software allows previously un-annotated genes to be discovered, and it also provides highly accurate alignment with known transcripts and an automated calculation of a confidence score for each variant identified [36]. Previously, NextGENe software was used for in-depth SNP analysis in genes associated with glioblastoma [37]. While searching for novel SNPs and variants in *MYH1*, we identified 10 SNPs that resulted deleterious amino acid substitutions for MYH1 protein in JNP and 3 such SNPs in Berkshire pigs, as compared with the reference sequence. Thus, the use of NextGENe might serve as a reliable and cost-effective approach for highly accurate genome-wide identification of DNA sequence variations.

Research demonstrated on the allelic variation of single genes that can be potential importance for genetic improvement of pork quality. In this study, we screened for functional coding-region genetic variants in *MYH1* using sequence- and structure-based algorithms, such as SIFT, PROVEAN, PolyPhen-2, I-Mutant3, and PANTHER. In our functional SNP analysis, SIFT and PROVEAN predicted that all the identified variants were deleterious. Concordant results of PolyPhen-2 and PANTHER predicted that 50 % of all identified variants were highly deleterious. Structure-based prediction performed using I-Mutant3.0 also showed that all identified amino acid substitutions were highly destabilizing. The results obtained using the *in silico* algorithm tools indicated that the L884T, K972C, N981G, and Q1285C variants were deleterious in JNP and H973G was deleterious in Berkshire pigs. Therefore, these nsSNP might be functionally involved in phenotypic traits in these pigs.

*In silico* analysis were performed using the HOPE tool indicated that misfolding and effects on molecular interactions of MYH1 can affect the protein’s structure and function. The mutated residues with the substitutions of L884T, K972C, N981G, and Q1285C possess specific sizes, charges, and hydrophobicity values, and the substitutions involve highly conserved residues in the wild-type protein. The mutated residues are located on the surface of a domain with as-yet unknown function, but the changes in charge are likely to disturb the ionic interactions of the protein; furthermore, interactions with other molecules or domains are also possibly affected by these mutations. For instance, changes in hydrophobicity caused by the mutations might affect multimerization.

By contrast, mutations in *MYH1* that alter gene expression can lead to disruption of muscle-specific biological and patho-physiological processes that control muscle growth and metabolism. The leucine at position 884 has been reported to be replaced with threonine in JNP. Leucine is a ketogenic amino acid that can be converted to acetyl-CoA and acetoacetate in muscle tissue; these intermediates can be used to synthesize fatty acids. Leucine supplementation has been documented to lead to an improvement in muscle color and intramuscular fat content in longissimus muscle [38]. Increased marbling or an elevated intramuscular fat content in meat is widely accepted to enhance the edibility of pork. Conversely, threonine is a major component of plasma γ-globulin in animals, and dietary threonine intake influences components of the immune system [39], which is reflected as an underlying expansion of immune surveillance in JNP [40].

The K972C mutation can also affect usual functions of MYH1 in JNP. During growth, lysine is critical for protein synthesis, muscle deposition, and carcass quality. A diet featuring a diminished content of lysine resulted in a substantial reduction in growth performance [41]. In this context, dietary lysine levels have been shown to regulate the expression of glucose transporter protein at the mRNA level [42]. Moreover, lysine is involved in cytokine synthesis and lymphocyte proliferation, and thus in the optimal functioning of the immune-system response to infection. By contrast, the reduced (Cys) and oxidized (Cys-Cys) forms of cysteine support animal growth equally well when supplemented in a cysteine-deficient and methionine-adequate diet. Investigation of the toxicity of cysteine in young pigs showed that 2 doses each of Cys and Cys-Cys resulted in markedly depressed weight gain, food intake, and weight gain: food intake ratio, regardless of the cysteine source [43].

Biochemical studies have revealed that glutamine, glutamate, proline, aspartate, asparagine, ornithine, citrulline, and arginine serve key regulatory functions in the immune response in pigs, and are inter convertible through complex inter-organ metabolism [39, 44]. It was demonstrated that asparagine is required to sustain maximal growth of young rats at different stages of growth [45]. Skeletal muscle is considered to oxidize certain branched-chain amino acids in muscle proteins during gluconeogenesis; these amino acids include aspartic acid, asparagine, glutamic acid, isoleucine, and valine [46]. The glycine was reported as nutritionally essential amino acid for maximal growth in young pigs [47]. Thus substitution of asparagine with glycine at residual position 981 in MYH1 might descend the intestinal health in neonates under conditions of oxidative stress.

Dietary supplementation with glutamine in early-weaned piglets prevents intestinal atrophy, and it also improves growth performance and meat quality in pigs. Investigation of the effects of glutamine on growth performance in piglets showed that this amino acid is extremely effective at improving growth performance [48]. Glutamine is the only amino acid in arterial blood that is taken up by the small intestine of pigs in the post-absorptive state. The conversion of glutamine into citrulline in the liver, kidney, and skeletal muscle is catalyzed by P5C and N-acetylglutamate synthase [44]. Glutamine is required for the synthesis of N-acetylglucosamine-6-phosphate, a common substrate for the synthesis of glycol-proteins that are highly enriched in intestinal mucosal cells [49]. Therefore, a mutation that changes glutamine to cysteine at position 1285 in MYH1 can suppress growth performance in the JNP breed. The diminished expression and abundant mutations in *MYH1* in the JNP breed can markedly influence skeletal muscle development. Interestingly, the food industry has been criticized in particular for the decline in the quality of pork obtained from lean breeds. The properties of lean meat are reported to be greatly influenced by the meat’s chemical composition [35, 50, 51]. Thus, the expression level and sequence of *MYH1* might contribute not only to the deficient growth performance but also the meat quality of the JNP breed.

GO analysis is used to characterize protein function and to elucidate trends in protein datasets [52]. Our previous study clearly showed that porcine MYH1 is primarily responsible for muscle contraction and muscle tissue development, and is the main structural constituent of porcine muscle [22]. Furthermore, an analysis of the biological process involving this structural muscle constituent showed a functional association of MYH1 with MYLPF. Protein-protein associations are interesting from multiple perspectives and are studied for diverse reasons, including for elucidating specific biological processes, enhancing our understanding of metabolic pathways, and deriving genotype-phenotype correlations [53]. The association of MYH1 with MYLPF was assessed using the STRING map view. Notably, *MYLPF* is a positional and functional candidate gene for meat production in domestic animals [8]. Phosphorylation of MYLPF, which is catalyzed by myosin light chain kinase in the presence of calcium and calmodulin (CaM), increases actin-activated myosin ATPase activity, and regulate contractile activity, which contributes to skeletal muscle energy metabolism and meat quality [10]. Strong interaction patterns observed for MYH1 and MYLPF indicated a combined role of these proteins in skeletal muscle development.

Elucidating the modulation of protein-protein associations is challenging, but is receiving increasing research attention [54]. Wang and Moult reported that protein-protein interactions, protein stability, and protein folding are influenced by nsSNP, which can therefore affect normal protein functions [55]. We used molecular docking and molecular dynamic approaches to study native and mutant MYH-MYLPF complexes in order to determine the effects of mutations on the interactions between the proteins. Compared with the native protein, the MYH1 mutants showed reduced interaction energy with MYLPF. Furthermore, the molecular dynamic analysis yielded certain insights into protein-protein interactions at the atomic level. RMSDs and RMSFs were calculated to illustrate the stability and flexibility of native and mutant MYH1-MYLPF complexes, and the results revealed that because of the incorporation of deleterious amino acids, the RMSD and RMSF values of the mutant complexes were lower and higher, respectively, than those of the native complex. An increase in protein stability can cause increased rigidity, whereas a reduction in the stability can cause an increase in the flexibility of a protein [56]. Thus, our analysis confirmed that the amino acid substitutions L884T, K972C, N981G, and Q1285C affect the stability and flexibility of the mutant complexes.

In summary, the results of our GO annotation, nsSNP analysis, QTL mapping and mRNA expression analysis of *MYH1* in JNP and Berkshire breeds indicate a role of this gene in muscle growth, meat quality and marbling, thus *MYH1*can serve as the candidate gene in relation to livestock production and/or meat quality of swine. Further, we have planned to carry out populations study for performance test to confirm the associations within and between the breeds and the degree of correlation that will guide us to include the identified nsSNP for breed improvement program.

## Conclusion

This study represents the first comprehensive investigation that has identified functional nsSNP in *MYH1*in JNP and Berkshire breeds using sequence- and structure-based homology algorithms. *In silico* annotation of certain nsSNP could explain the functional effects of these mutations. Furthermore, pathway-based analysis of protein-protein interactions highlighted the importance of the interaction between MYH1 and MYLPF in skeletal muscle development. The results of this study suggest that the variants L884T, K972C, N981G, and Q1285C in MYH1 in JNP might represent a cause for the poor growth performance of this breed. Thus, these MYH1 variants might be useful as selection markers for improving growth performance in the JNP breed.

## Methods

### Ethics statement and animal housing

The study was conducted under strict accordance with the recommendations in the guide for the care and use of animals of the Animal Bioethics Committee (permit number: 2013–0009) of Jeju National University, Jeju-Si, Jeju-Do, Republic of Korea. The animals were handled accordance with proper animal welfare guidelines [57]. Pure-breed adult female animals from JNP and Berkshire breeds (*n* = 5 from each breed; average individual body weight, 84.76 ± 3.5 kg) were reared under the same environmental and nutritional conditions. All the pigs were provided *ad libitum* access to commercial feed (Seoul Feed, Jeju-Si, South Korea) and water. Pigs were housed in concrete-floored pens that contained a nipple-bowl drinker and a feeder. Animals were sacrificed through entailed exsanguination following electric stunning, with all possible effort being devoted to minimize suffering.

### Sample preparation for RNA-seq using Illumina HiSeq

The *longissimus dorsi* muscle samples were collection from the total number of 10 animals (*n* = 5 from each of JNP and Berkshire breeds) and immediately submerged in RNAlater (Sigma-Aldrich) followed by the frozen in liquid nitrogen. Total RNA from the muscle samples were extracted using TRIzol (Invitrogen Life Technologies, Carlsbad, CA). The RNA integrity has been verified onto a 0.8 % agarose gel and evaluating the 28S and 18S ribosomal RNA bands. The purity and RNA concentration was measured with a BioPhotometer (Eppendorf). Concentrations for muscle samples ranged between 94–138 ng/μl (total yield 1.85–2.40 μg RNA). Approximately, 1 μg of RNA from each sample converted to cDNA in the subsequent 20 μl reaction using Superscript III reverse transcriptase with oligo dT. The second strand of cDNA was according to the manufacturer’s recommended protocol (Illumina, San Diego, CA, USA). The cDNA was diluted with nuclease free water and stored at −20 °C and further used for RNA-seq libraries prepared.

The TruSeq DNA Sample Prep. Kit (Illumina, San Diego, CA) was used for library construction following the manufacturer’s guidelines. RNA sequencing has been performed on the Illumina HiSeq 2000 platform. The fastQC software was used to perform a quality check on raw sequence data. Paired-end sequence reads were mapped to the pig reference genome (*S. scrofa* 10.2) from the Ensembl database with default settings using Bowtie2.

### Retrieval of SNPs in *MYH1*of JNP and Berkshire breeds

**Fig. 4:**
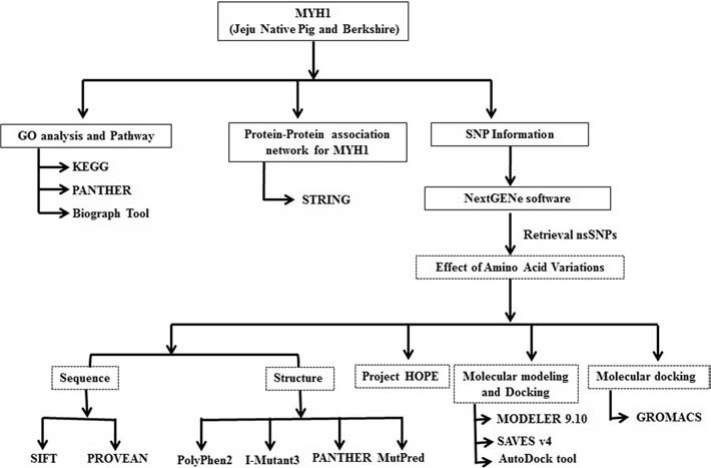
The schema of the semantics *in silico* analysis of the structural and functional assessment of MYH1. *In silico* methods were used carefully to evaluate the ontology of *MYH1* gene function, protein association network for MYH1 and the effects of the nsNSPs on the MYH1 functions

The mapping of sequenced transcriptome fragments on to the reference sequence is particularly important for identification of polymorphisms [58]. Hence, the NextGENe V 2.3.4.5 (SoftGenetics LLC, State College PA, USA) software allowed us to identify variants of *MYH1* in JNP and Berkshire using RNA-seq data. Data on *MYH1*from *S. scrofa* were collected from Entrez Gene in the National Center for Biotechnology Information (NCBI) database. SNP information for *MYH1* was retrieved from NCBI dbSNP (http://www.ncbi.nlm.nih.gov/snp); Ensembl genome browser 72: *Sus scrofa* (http://asia.ensembl.org/Sus_scrofa/Info/Index) and compiled from experimental data. Figure 4 illustrated the various *in silico* methods associated with structural and functional assessment of porcine *MYH1* in our study.

### Gene ontology (GO) of *MYH1* for function and pathway analysis

GO analysis was used to study the molecular, biological, and cellular processes associated with *MYH1* function in *S. scrofa*. Functional classification views of *MYH1*were obtained using the PANTHER classification system (http://www.pantherdb.org/). Furthermore, the pathways of *MYH1* were analyzed using the Kyoto Encyclopedia of Gene and Genomics (KEGG) tool (http://www.genome.jp/kegg/pathway.html). The Biograph tool (http://www.biograph.be) and ToppGene suite (https://toppgene.cchmc.org//) were used for in-deep graphical representation of biological processes and gene pathway analysis, respectively.

### Quantitative analysis of *MYH1* expression

The reverse transcription PCR (RT-PCR) was carried out through using 2.5 μl of cDNA. Each reaction included a positive porcine genomic DNA control, a negative control and a no-template control. The primers were designed using the online program Primer-3 (Table 4). The NCBI database was used to search for available porcine *MYH1* and *GAPDH* sequences in order to design primers.

**Table 4:** List of the primers sequences used for qRT PCR

Genes name	CDS region	Primer sequences	Annealing temperature (Ta)	Product size	Genebank IDs
*MYH1*	42….5861	F 5′ AAGGGACTGTCCAGAGCAGA 3′	55.0 °C	225	NM_001104951.1
		R 5′ CACAGAAGAGGCCCGAGTAG 3′			
*GAPDH*	101….1102	F 5′ AGAAGGTGGTGAAGCAGG 3′	61.0 °C	170	NM_001206359.1
		R 5′GTCGTACCAGGAAATGAGC3′			

Differential expression of *MYH1* was verified by quantitative real-time PCR (RT-qPCR) on the 10 animals from two breeds previously selected. The experiment setup and the descriptions for RT-qPCR analysis were explained according to MIQE guideline in this study [59]. Transcripts from each individual pig were amplified and detected using EvaGreen dye (Biotium, Hayward, CA, USA). The qRT-PCR was performed using an Applied Biosystems Step-One PCR system in a 10 μl reaction volume with 200 μM of each primer set. Each individual sample was quantified in triplicate under the following amplification conditions: 95 °C for 10 min, followed by 40 cycles of 95 °C for 30 s and 60 °C for 1 min. Standard curve methods were used to define the efficiency of real-time PCR. The efficiency of amplification of the target gene was compared with that of the endogenous *GAPDH* control transcript. Samples that did not include reverse transcriptase were included as negative controls to monitor DNA contamination, and four blank samples were added as qPCR negative controls.

Further, the western blotting assay was performed for expression profiling of MYH1 in JNP and Berkshire. For blotting assay, we used sixty micrograms of protein extract are diluted with 1:1with 2X loading buffer as described in our previous study [60] and loaded on 12 % polyacrylamide gel. Proteins were separated. Protein concentrations were calculated by using Pierce BCA Protein Assay kit (Thermo Scientific) following the manufacturer’s instructions. After electrophoresis the protein sample of MYH1 and β-actin were transferred to nitrocellulose membrane and blocked for 2 h at room temperature. All antibodies were purchased from Santa Cruz Biotechnology, Inc., CA, USA. The membrane was incubated with primary antibody for MYH1 (Mouse monoclonal. 1:50; catalogue no. sc 53055) with the control β actin (Mouse polyclonal, 1:500; catalogue no. sc-2025) for overnight following by treated with secondary antibodies (Rabbit anti mouse, 1:1000; catalogue no. sc-358922) for one hour. The expression of protein was detected by specific chemiluminescence detection kit using Luminescent Image Analyzer (LAS-4000mini) (GE Healthcare, NJ, USA). The means were compared between Berkshire and JNPs. The relative band intensity of MYH1 normalized with relative to the band intensities of β-actin using Image J software (National Institute of Health, Bethesda, Maryland, USA).

### Data mining for QTLs in porcine *MYH1* region

QTL analysis was conducted to map *MYH1* region whose expression significantly correlated to quantitative traits in commercial pig breeding schemes. QTLs analysis is based on the linkage between markers and QTL. The publicly available Animal QTL database for pig (http://www.animalgenome.org/cgi-bin/QTLdb/SS/index) is used for compare, confirm, and locate the most plausible location for the gene of interest associated with quantitative traits. Taking into account previously observations we highlight QTL located on the SSC12 (57978043….57981608) region on the reliability of QTL map using JavaScript-based genome browser or JBrowse.

### Functional concatenation of amino acid substitutions for *MYH1*in JNP and Berkshire breeds

The missense variants obtained from NextGENe application were proposed for further verification. The open-source tools were employed for sequence- and structure-based approaches to predicate the non-synonymous SNP. The deleterious score of the substituted amino acids that alter protein function(s) was predicated by SIFT (Sorting Intolerant from Tolerant; http://sift.jcvi.org/www/SIFT_seq_submit2.html) tool used homologous sequences. We submitted the amino acid sequence of porcine MYH1 as query obtained from the NCBI database. The output SIFT scores were classified as damaging when they ranged from 0.00 to 0.05; scores between 0.051 and 0.10 were considered potentially damaging; and 1.00 was considered neutral. Here, when scores were below the threshold fixed at 0.05, the relevant amino acid substitutions were predicted to be deleterious. Further, we used online tool PROVEAN (Protein Variation Effect Analyzer; http://provean.jcvi.org/seq_submit.php) to predict the possible impact of a substituted amino acid on protein structure and function. A protein variant was considered deleterious if the final score was below the threshold score of −2.5; scores above this threshold were considered neutral.

PolyPhen-2 (Polymorphism Phenotyping v2; http://genetics.bwh.harvard.edu/pph2/) was used for predicting the possible impact of amino acid substitutions on protein structure and function by using straightforward physical and evolutionary comparative considerations [61]. The protein sequence, position of the substituted amino acid, and variation details were used as the input options for PolyPhen-2. The scores were classified as probably damaging (≥2), possibly damaging (1.50 − 1.99), potentially damaging (1.25 − 1.49), and benign (0.00 − 0.99).

### Identification of potential consequences of nsSNP in *MYH1* in JNP and Berkshire breeds by using PANTHER

We used PANTHER (http://pantherdb.org/tools/csnpScoreForm.jsp) to accurately and robustly analyze the effect of mutations on protein functions. In PANTHER, multiple sequence alignments and hidden Markov model (HMM)-based statistical modeling are used for the evolutionary analysis of a given amino acid at a particular position in the protein of interest. The method can generate position-specific evolutionary conservation scores (PSECs), and the substitution PSEC (subPSEC) score is calculated from alignments to HMMs in the PANTHER database in order to separate neutral from deleterious SNPs. If a protein’s subPSEC value is 0, the substitution is interpreted as being functionally neutral; values that are ≤ −3 are considered deleterious.

### Using I-Mutant3 to predict the effect of mutations on MYH1 protein stability in JNP and Berkshire breeds

I-Mutant 3.0 (http://gpcr2.biocomp.unibo.it/cgi/predictors/I-Mutant3.0/I-Mutant3.0.cgi) is support vector machine-based tools used for analyze protein stability. As the input query, we used the FASTA sequence of porcine MYH1protein obtained from NCBI and the amino acid substitutions and their respective positions in JNP and Berkshire breeds. This method can be used to predict whether a mutation’s effect on a protein is largely destabilizing (<−0.5 Kcal mol^−1^), largely stabilizing (>0.5 Kcal mol^−1^), or weak (−0.5 ≤ DDG ≤ 0.5 Kcal mol^−1^). This value is calculated after subtracting the value of the unfolding Gibbs free energy of the wild-type protein from the corresponding value of the mutant protein.

### Computational identification of mutational effects on protein function by using MutPred

The web-based tool MutPred (http://mutpred.mutdb.org/) was used for classifying amino acid substitutions as being disease-associated or neutral. MutPred is based on SIFT and a gain or loss of 14 different structural and functional properties [62]. We used the nsSNP from the JNP and Berkshire trials as input. The MutPred output consists of a general score associated with the deleterious effect of the substituted amino acid and the top-5 property scores (P). The “*P*-values” in MutPred are classified as actionable hypothesis (*P* < 0.05), confident hypothesis (*P* < 0.05), and very confident hypothesis (*P* < 0.01).

### Predication of mutational effect on MYH1 using HOPE

Mutations located in the protein-coding regions of a genome can be affect on it structure and functions. Knowledge of the effect of these mutations on the three-dimensional (3D) structure of a protein provides mechanistic insights into the protein’s functions and also enhances our understanding of substantial changes in the protein and can aid in the design of further experiments [35, 63]. HOPE (Have yOur Protein Explained; http://www.cmbi.ru.nl/hope/) is a next-generation web-based tool for automated mutant analysis. HOPE collects information from databases such as the 3D structure of proteins and protein sequences from the UniProt database that can be used to process these data and to predict the effects of a mutation on the 3D structure and function of a protein. The protein sequence of *MYH1* and 4 most deleterious residual variants in JNP were used as the input in HOPE. The output report was focused on the effect of the mutations on protein structure.

### Association network of MYH1with relevant functional proteins

All protein-encoding genes can be grouped and organized in genome through protein-protein associations [64]. A graphical representation of protein-protein association by using a network provides a high-level view of functional associations and facilitates analysis of the modulation of interactions with partner proteins in biological processes. The STRING (Search Tool for the Retrieval of Interacting Genes/Protein; http://string-db.org/) database was used to assemble, evaluate, and disseminate information regarding the structural, functional, and evolutional properties of proteins. STRING integrates and ranks these associations under a common framework. These data are weighted and integrated and then a confidence score is calculated for all protein interactions. The results of various computational predictions can be inspected from different designated views. Here, the closest associated protein was selected for a docking study.

### Molecular modeling and validation of the model

The protein structure of MYH1 was modeled to compare the structural stability of the native and mutant proteins. The protein structure of MYH1 from *S. scrofa* is not yet available in Protein Data Bank (PDB); therefore, we used MODELER 9.10 and modeled the structure of MYH1 by using the sequence available in the NCBI database. Sequences homologous to the target structure were retrieved by performing Psi-BLAST against the PDB database. Template sequences exhibiting the highest similarity to the target sequence were identified and considered for modeling studies. The obtained model was further subjected to structural validation. PyMOL was used for visualizing the model, calculating electrostatic potentials, and generating images. Moreover, we modeled the 3D homologous structures of the proteins that are closely associated with *S. scrofa* MYH1 by using MODELER 9.10. The SAVES v4 (Structure Analysis and Verification Server; http://services.mbi.ucla.edu/SAVES/) was used to evaluate the internal consistency and reliability of the homologous models. The Psi/Phi Ramachandran plot was generated using PROCHECK.

### Molecular docking study

The modeled structures of native and mutant MYH1 proteins were analyzed for molecular docking with the structure obtained for the *S. scrofa* MYLPF protein. Docking simulation was performed by using the AutoDock tool. Docking was optimized by using the Lamarckian genetic algorithm (LGA) for 70,000 rotations, and the population size was set to 150. The obtained confirmations were then summarized, collected, and extracted using AutoDock tools.

### Molecular dynamic simulation

The PDB files obtained from the docking study were used as an input for molecular dynamic simulation performed. Protein complexes were solvated in a cubic water box. The topology and coordinate files were generated using GROMACS server (GROningen MAchine for Chemical Simulations; http://www.gromacs.org/). Charges were neutralized by adding Cl^−^ ions. Temperature is frequently equilibrated under constant number, volume, and temperature (NVT) ensemble. Considering our computational power, 25 ns of equilibration with position restraints of the temperature on the protein was set at 300 K. Desired pressures were supplied after the thermal equilibration in order to acquire the proper density. The trajectory analysis performed using molecular dynamic simulations allowed us to understand the stability of the system. We applied periodic boundary conditions to generate the final trajectory files, which were analyzed in order to obtain the root-mean-square deviation (RMSD) and root-mean-square fluctuation (RMSF).

### Statistical analysis and data processing

Haplotype blocks and linkage disequilibrium (LD) plots have been constructed using Haploview version 4.2 with the default algorithm (www.broad.mit.edu/mpg/haploview). After removing outliers (Grubb’s test) by dividing the covariance of the data sets with the product of their standard deviations the Pearson’s correlation coefficient (*r*) was calculated.

The relative quantitative (RQ) of *MYH1* expression was studied by means ± SEM of 5 animals with triplicates (*P* < 0.05). To correct for technical inter-run variation among triplicate reactions of the same sample measured in different runs, the data were calibrated by calculating the average cycle threshold (Ct) value over all the samples in each run [65, 66]. After the calibration, the average Ct-value of each triplicate reaction was converted to relative quantities and these were analyzed using Tukey’s b test.

## Availability of supporting data and materials

The checklist of "ARRIVE Guidelines  for Animal Research: Reporting In Vivo Experiments" for the present study was provided in supplementary data (Additional file 2). The gene differential expression analyses between the Berkshire and JNP at two different development stages (adult and piglet) are freely available in Biopop database at Seoul National University (http://biopopdb.snu.ac.kr/PIG_DEG/). The software for RNA-seq analysis and mapping used in this study includes NextGENe V2.0 (http://www.softgenetics.com/NextGENe.html), TopHat (https://ccb.jhu.edu/software/tophat/index.shtml) [67]. SNP information used in this study can be found from dbSNP (http://www.ncbi.nlm.nih.gov/snp/) [68] and Ensembl genome browser 72: *Sus scrofa* (http://asia.ensembl.org/Sus_scrofa/Info/Index) [69]. The Gene ontology and pathway analysis were performed using PANTHER classification system (http://www.pantherdb.org/) [70], Kyoto Encyclopedia of Gene and Genomics (http://www.genome.jp/kegg/pathway.html) [71], Biograph tool (http://www.biograph.be) [72] and ToppGene suite (https://toppgene.cchmc.org///) [73]. The primers were designed using Primer-3 (http://primer3.ut.ee/) [74]. The QTL data can be downloaded from PigQTLdb at http://www.animalgenome.org/cgi-bin/QTLdb/SS/index [75]. The tools used for predication of sequential and structural missense variation are includes SIFT (http://sift.jcvi.org/www/SIFT_seq_submit2.html) [76], PolyPhen-2 (http://genetics.bwh.harvard.edu/pph2/) [77], PANTHER (http://pantherdb.org/tools/csnpScoreForm.jsp) [70], I-Mutant 3.0 (http://gpcr2.biocomp.unibo.it/cgi/predictors/I-Mutant3.0/I-Mutant3.0.cgi) [78], MutPred (http://mutpred.mutdb.org/) [79] and HOPE (Have yOur Protein Explained; http://www.cmbi.ru.nl/hope/) [63]. Protein-protein association network was viewed using STRING at http://string-db.org/. The 3D homologous models and the validation were performed using MODELER (https://salilab.org/modeller/) [80] and SAVES v4 at http://services.mbi.ucla.edu/SAVES. Molecular docking and dynamic simulation were performed using AutoDock tool at http://autodock.scripps.edu/resources/adt and GROMACS server at http://www.gromacs.org. Haplotype blocks and linkage disequilibrium plots have been constructed using Haploview version 4.2 (www.broad.mit.edu/mpg/haploview) [81].

## Additional files

Additional file 1:
**Figure S1.** Graphical views of the GO related to the muscle growth and development of porcine *MYH1*. (A) The biological process of muscle contraction. (B) The biological process of muscle organ development. **Figure S2.** The significant pathways terms of *MYH1* gene in porcine. **Figure S3.** The scatter plot of differentially expressed genes and QTLs map in *MYH1* region of SSC12. (A) Scatter plots was constructed with log2- for the visual comparison of gene expression levels in muscle tissue samples of JNP and Berkshire. The CNR scores and position of *MYH1* gene demonstrated with in the box. (B) The position of most significant QTLs those are associated with meat and carcass quality traits reported in *MYH1* region. **Figure S4.** The 3D homologous model of *MYH1*. The tertiary structure of native *MYH1* is drawn here as a cartoon model. **Figure S5.** Ramachandran Plot of native *MYH1*. The red, brown, and yellow regions represent favored, allowed, and “generously allowed” regions as defined by ProCheck. **Figure S6.** The 3D homologous model of MYLPF is drawn here as a cartoon model. **Figure S7.** Ramachandran Plot of MYLPF represents the favored, allowed, and “generously allowed” regions as defined by ProCheck for MYLPF. **Figure S8.** The RMSD values of all backbone atoms of MYH1- MYLPF protein complexes. The RMSD (in nanometer) is at ordinate and Time (in nano-second) at abscissa. The structural simulations for the native *MYH1* (green), mutant *MYH1* L884T (blue), K972C (black), N981G (pink) and Q1285C (red) complexes with MYLPF. **Table S1.** Ontology of *MYH1* gene of *Sus scrofa*. **Table S2.** Pathways of *MYH1* gene of *Sus scrofa*. **Table S3.** Prediction of the mutational effects. Prediction of the mutational effects on the function on *MYH1* protein from JNP and Berkshire using MutPred. **Table S4.** Predication the functional partners of protein-protein interactions from STRING database. (DOCX 966 kb) Additional file 2:
**The ARRIVE Guidelines Checklist Animal Research: Reporting In Vivo Experiments.** (PDF 386 kb)

## Abbreviations

ACTG1: actin, cytoplasmic 1
CaM: calmodulin
CGN: cingulin
CNR: copy number ration
Ct-value: cycle threshold value
GO: gene ontology
GROMACS: GROningen MAchine for chemical simulations
HOPE: have your protein explained
KEGG: kyoto encyclopedia of gene and genomics
LD: linkage disequilibrium
LGA: lamarckian genetic algorithm
MYH1: myosin heavy chain 1
MYL1: myosin, light chain 1, alkali; skeletal, fast
MYL6: myosin, light chain 6
MYLPF: myosin light chain phosphorylatable fast
NGS: next generation sequencing
nsSNP: non-synonymous SNP
PDB: protein data bank
PolyPhen-2: polymorphism phenotyping v2
PROVEAN: protein variation effect analyzer
qRT-PCR: quantitative real time PCR
QTL: quantitative trait loci
RHOA: Ras homolog gene family, member A
RMSD: root mean square deviation
RMSF: root mean square fluctuation
RQ: relative quantitative
RT-PCR: reverse transcription PCR
SAVES: structure analysis and verification server
SIFT: sorting intolerant from tolerant
SSC: *Sus scrofa* chromosome
STRING: search tool for the retrieval of interacting genes/protein
TNNC2: troponin C type 2
TNNI2: Troponin I type 2 (skeletal, fast)
TNNI3: Troponin I, cardiac muscle
TTN: Titin
