# Supplementary material for: An integrated in silico approach for functional and structural impact of non- synonymous SNPs in the MYH1 gene in Jeju Native Pigs
Source: BMC Genet. 2016 Feb 4;17:35. doi: 10.1186/s12863-016-0341-1 (PMC4741023; doi:10.1186/s12863-016-0341-1)
Supplement: Additional file 1: — Figure S1. Graphical views of the GO related to the muscle growth and development of porcine MYH1. (A) The biological process of muscle contraction. (B) The biological process of muscle organ development. Figure S2. The significant pathways terms of MYH1 gene in porcine. Figure S3. The scatter plot of differentially expressed genes and QTLs map in MYH1 region of SSC12. (A) Scatter plots was constructed with log2- for the visual comparison of gene expression levels in muscle tissue samples of JNP and Berkshire. The CNR scores and position of MYH1 gene demonstrated with in the box. (B) The position of most significant QTLs those are associated with meat and carcass quality traits reported in MYH1 region. Figure S4. The 3D homologous model of MYH1. The tertiary structure of native MYH1 is drawn here as a cartoon model. Figure S5. Ramachandran Plot of native MYH1. The red, brown, and yellow regions represent favored, allowed, and “generously allowed” regions as defined by ProCheck. Figure S6. The 3D homologous model of MYLPF is drawn here as a cartoon model. Figure S7. Ramachandran Plot of MYLPF represents the favored, allowed, and “generously allowed” regions as defined by ProCheck for MYLPF. Figure S8. The RMSD values of all backbone atoms of MYH1- MYLPF protein complexes. The RMSD (in nanometer) is at ordinate and Time (in nano-second) at abscissa. The structural simulations for the native MYH1 (green), mutant MYH1 L884T (blue), K972C (black), N981G (pink) and Q1285C (red) complexes with MYLPF. Table S1. Ontology of MYH1 gene of Sus scrofa. Table S2. Pathways of MYH1 gene of Sus scrofa. Table S3. Prediction of the mutational effects. Prediction of the mutational effects on the function on MYH1 protein from JNP and Berkshire using MutPred. Table S4. Predication the functional partners of protein-protein interactions from STRING database. (DOCX 966 kb) [file 12863_2016_341_MOESM1_ESM.docx]

**Additional File 1**

**(This file contains the Supplementary Tables and Figures)**

**An integrated *in silico* approach for functional and structural impact of non- synonymous SNPs in the *MYH1* gene in Jeju Native Pigs**

Mrinmoy Ghosh^1^, Simrinder Singh Sodhi^1^, Neelesh Sharma^2^, Raj Kumar Mongre^1^, Nameun Kim^1^, Amit Kumar Singh^1^, Sung Jin Lee^3^, Dae Cheol Kim^4^, Sung Woo Kim^5^, Hak Kyo Lee^6^, Ki-Duk Song^6*^ and Dong Kee Jeong^1*^

1. Department of Animal Biotechnology, Faculty of Biotechnology, Jeju National University, Jeju-Do 690-756, Republic of Korea
2. Sher-e-Kashmir University of Agricultural Sciences and Technology, R.S. Pura, Jammu, India
3. Department of Animal Biotechnology, College of Animal Bioscience and Technology, Kangwon National University, Chuncheon 200-701, Republic of Korea
4. Livestock Promotion Institute, Jeju Special Self-governing Province, Jeju-Do 690-756Republic of Korea.
5. Animal Genetic Resources Station, National Institute of Animal Science, Rural Administration, Namwon, Republic of Korea
6. Department of Animal Biotechnology, Chonbuk National University, Jeonju, 561-756, Republic of Korea

*Correspondence

Dong Kee Jeong

E mail: dkjeong@jejunu.ac.kr

Ki-Duk Song

E mail: kiduk.song@gmail.com

**Table S1 Ontology of *MYH1* gene of *Sus scrofa***

| **GO biological process** | **GO molecular function** | **GO cellular component** |
| --- | --- | --- |
| Cytokinesis (GO:0000910) | Motor activity (GO:0003774) | Plasma membrane (GO:0005886) |
| Cellular component movement (GO:0006928) | Structural constituent of cytoskeleton (GO:0005200) | Cell junction (GO:0030054) |
| Mitosis (GO:0007067) | Protein binding (GO:0005515) | Actin cytoskeleton (GO:0015629) |
| Cell communication (GO:0007154) | Enzyme regulator activity (GO:0030234) | Intracellular (GO:0005622) |
| Muscle contraction (GO:0006936) |  |  |
| Sensory perception of sound (GO:0007605) |  |  |
| Sensory perception (GO:0007600) |  |  |
| Mesoderm development (GO:0007498) |  |  |
| Cellular component morphogenesis (GO:0032989) |  |  |
| Muscle organ development (GO:0007517) |  |  |
| Intracellular protein transport (GO:0006886) |  |  |
| Intracellular protein transport (GO:0006886) |  |  |
| Vesicle-mediated transport (GO:0016192) |  |  |
| Regulation of catalytic activity (GO:0050790) |  |  |
| Cellular component organization (GO:0016043) |  |  |

**Table S2 Pathways of *MYH1* gene of *Sus scrofa***

| **Gene** | **Pathways** |
| --- | --- |
| *MYH1* | - Inflammation mediated by chemokine and cytokine signaling pathway-> Myosin - Wnt signaling pathway-> NFAT Target Genes - Dysregulate signaling pathways - Nicotinic acetylcholine receptor signaling pathway->Myosin - Cytoskeletal regulation by Rho GTPase->Myosin light chain - Epithelial tight junctions - Translocation of GLUT4 to the Plasma Membrane |

**Table S3** **Prediction of the mutational effects.** The effect of the mutation on the function of *MYH1* protein from JNP and Berkshire using MutPred

| **Mutation** | **Probability of deleterious mutation** | **Probable Top 5 functional disruptions** |
| --- | --- | --- |
| JNP | | |
| M881R | 0.476 | Gain of MoRF binding (P = 0.029), Loss of catalytic residue at M881 (P = 0.0338), Gain of methylation at M881 (P = 0.0373), Loss of ubiquitination at K880 (P = 0.3072), Loss of glycosylation at K880 (P = 0.3324) |
| L884T | 0.534 | Loss of stability (P = 0.0052), Gain of phosphorylation at L884 (P = 0.1062), Gain of MoRF binding (P = 0.2095), Loss of ubiquitination at K888 (P = 0.2547), Gain of glycosylation at K880 (P = 0.3615) |
| A987T | 0.233 | Gain of phosphorylation at A987 (P = 0.0584), Loss of stability (P = 0.1501), Gain of solvent accessibility (P = 0.3819), Gain of MoRF binding (P = 0.3948), Gain of helix (P = 0.6868) |
| K970A | 0.193 | Loss of ubiquitination at K970 (P = 0.0141), Loss of methylation at K970 (P = 0.0496), Loss of solvent accessibility (P = 0.1202), Loss of phosphorylation at T975 (P = 0.177), Loss of glycosylation at K970 (P = 0.2003) |
| K972C | 0.582 | Loss of ubiquitination at K972 (P = 0.0134), Loss of methylation at K972 (P = 0.0245), Loss of phosphorylation at T975 (P = 0.1101), Gain of catalytic residue at H973 (P = 0.1599), Loss of glycosylation at K972 (P = 0.2148) |
| H973G | 0.227 | Loss of ubiquitination at K970 (P = 0.1223), Loss of methylation at K972 (P = 0.1405), Gain of phosphorylation at T975 (P = 0.2086), Gain of glycosylation at K972 (P = 0.2696), Loss of solvent accessibility (P = 0.3103) |
| T975V | 0.328 | Loss of phosphorylation at T975 (P = 0.0362), Gain of ubiquitination at K972 (P = 0.1028), Loss of methylation at K972 (P = 0.1166), Gain of MoRF binding (P = 0.3338), Loss of glycosylation at K970 (P = 0.4649) |
| N981G | 0.506 | Loss of stability (P = 0.0434), Gain of glycosylation at K980 (P = 0.1303), Loss of methylation at K978 (P = 0.1576), Gain of phosphorylation at T983 (P = 0.2989), Gain of ubiquitination at K978 (P = 0.31) |
| Q1285C | 0.570 | Gain of methylation at R1283 (P = 0.0872), Loss of MoRF binding (P = 0.0907), Gain of catalytic residue at R1283 (P = 0.1868), Loss of phosphorylation at S1288 (P = 0.3329), Loss of solvent accessibility (P = 0.4141) |
| T1286P | 0.470 | Loss of phosphorylation at T1286 (P = 0.0171), Loss of MoRF binding (P = 0.0891), Loss of methylation at R1283 (P = 0.1353), Gain of glycosylation at S1288 (P = 0.1372), Loss of stability (P = 0.5542) |
| Berkshire | | |
| A897T | 0.345 | Gain of phosphorylation at A897 (P = 0.08), Loss of stability (P = 0.377), Gain of solvent accessibility (P = 0.3819), Loss of glycosylation at S901 (P = 0.63), Loss of MoRF binding (P = 0.647) |
| H973G | 0.227 | Loss of ubiquitination at K970 (P = 0.1223), Loss of methylation at K972 (P = 0.1405), Gain of phosphorylation at T975 (P = 0.2086), Gain of glycosylation at K972 (P = 0.2696), Loss of solvent accessibility (P = 0.3103) |

**Table S4 Predication the interaction score of proteins.** Predicted functional partners of protein-protein interactions from STRING database.

| **Associated Proteins** | **Full name** | **Score** |
| --- | --- | --- |
| MYLPF | Myosin light chain, phosphorylatable, fast skeletal muscle | 0.858 |
| MYL6 | Myosin, light chain 6, alkali, smooth muscle and non-muscle | 0.840 |
| ACTG1 | Actin, cytoplasmic 1 (Beta-actin) | 0.800 |
| RHOA | Ras homolog gene family, member A | 0.796 |
| CGN | Cingulin | 0.728 |
| TNNI3 | Troponin I, cardiac muscle | 0.727 |
| TNNC2 | Troponin C type 2 | 0.721 |
| MYL1 | Myosin, light chain 1, alkali; skeletal, fast | 0.721 |
| TNNI2 | Troponin I type 2 (skeletal, fast) | 0.678 |
| TTN | Titin | 0.677 |

**Additional Figures:**

**
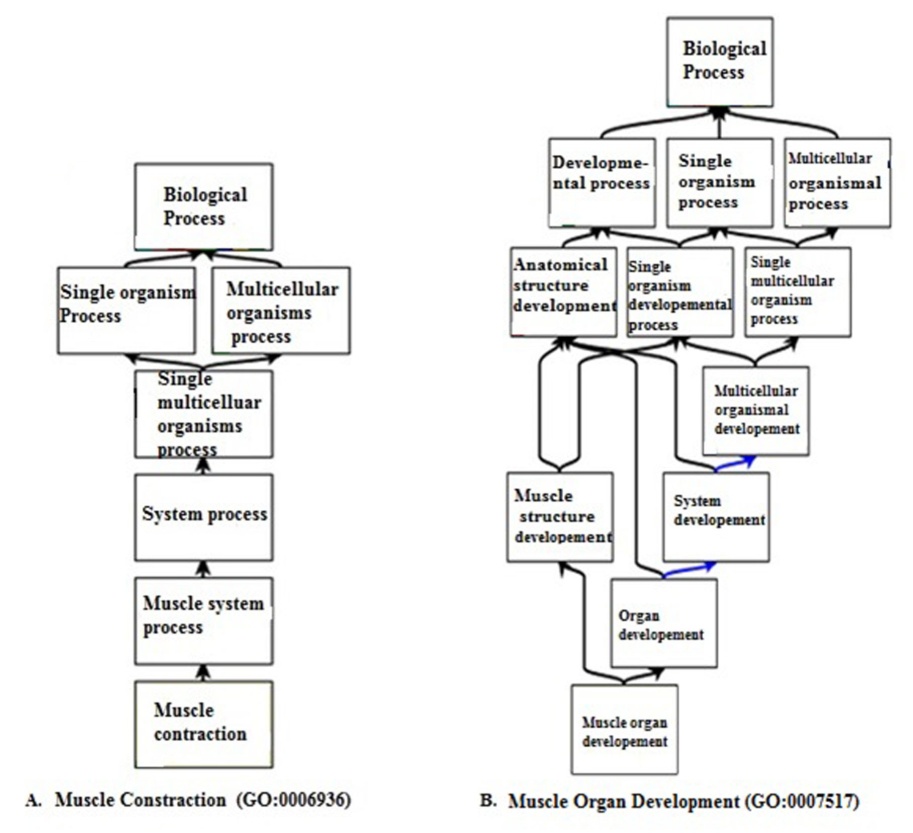
**

**Figure S1 Graphical views of the GO related to the muscle growth and development of porcine *MYH1***. A) The graphical representation of the biological process of muscle contraction. B) The graphical representation of the biological process of muscle organ development.

**
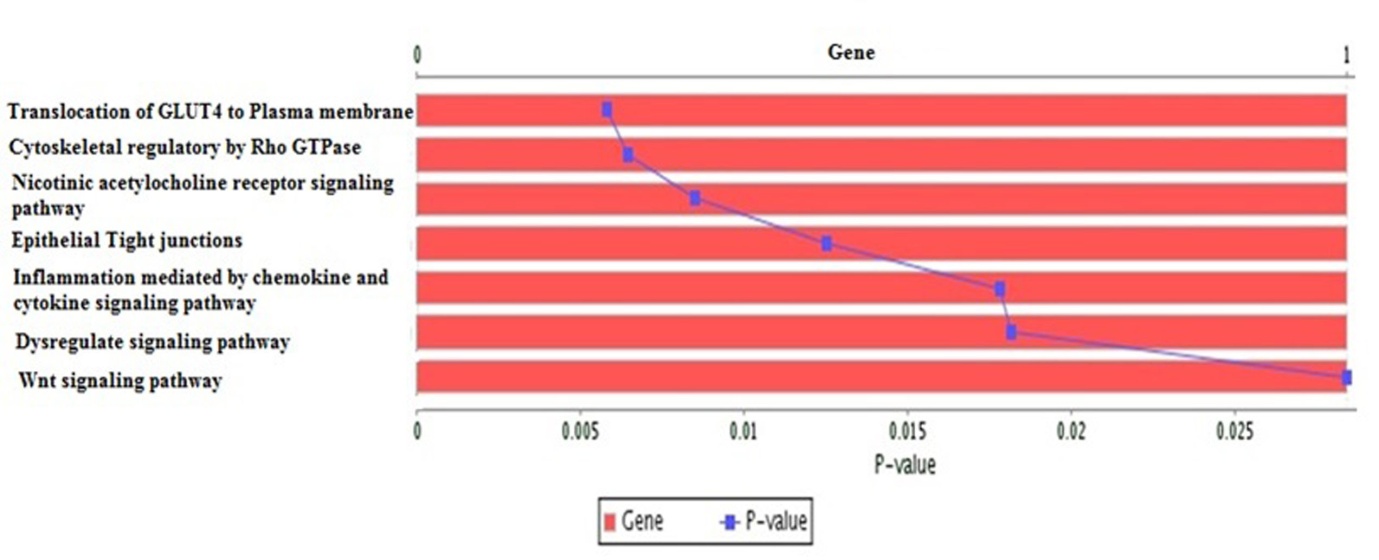
**

**Figure S2 The pathways of MYH1 gene**. The significant terms of the pathways of porcine *MYH1* gene.


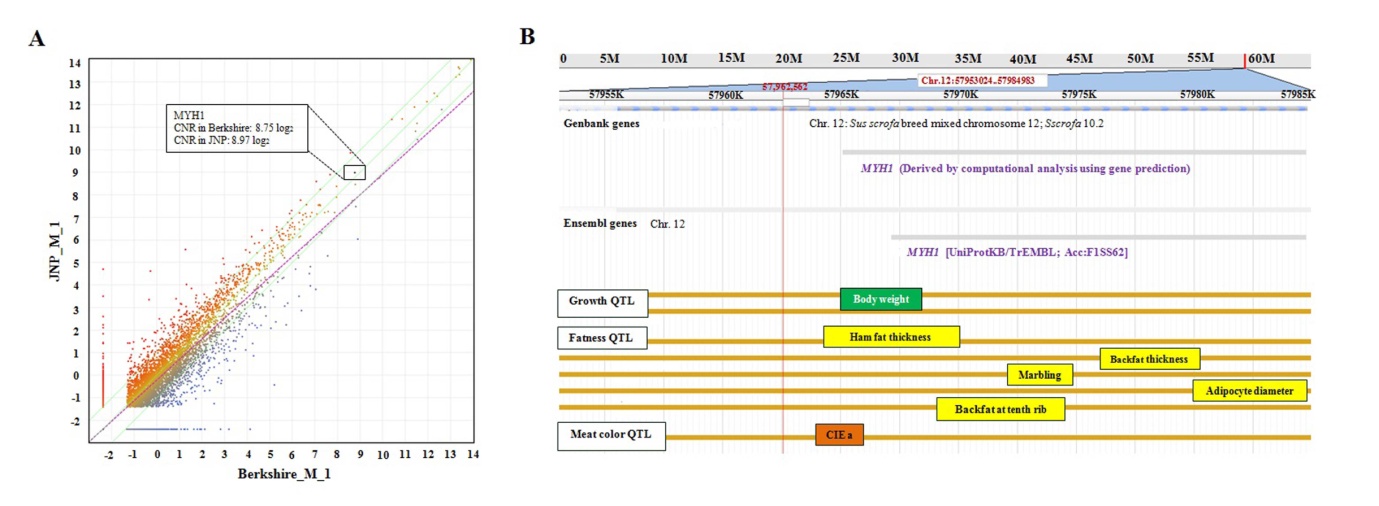


**Figure S3 The scatter plot of differentially expressed genes and QTLs map in *MYH1* region of SSC12.** (A) Scatter plots was constructed with log2- for the visual comparison of gene expression levels in muscle tissue samples of JNP and Berkshire. Each data point on the plot represents an individual gene. The CNR scores and position of *MYH1* gene demonstrated with in the box. (B) The position of most significant QTLs those are associated with meat and carcass quality traits reported in *MYH1* region.


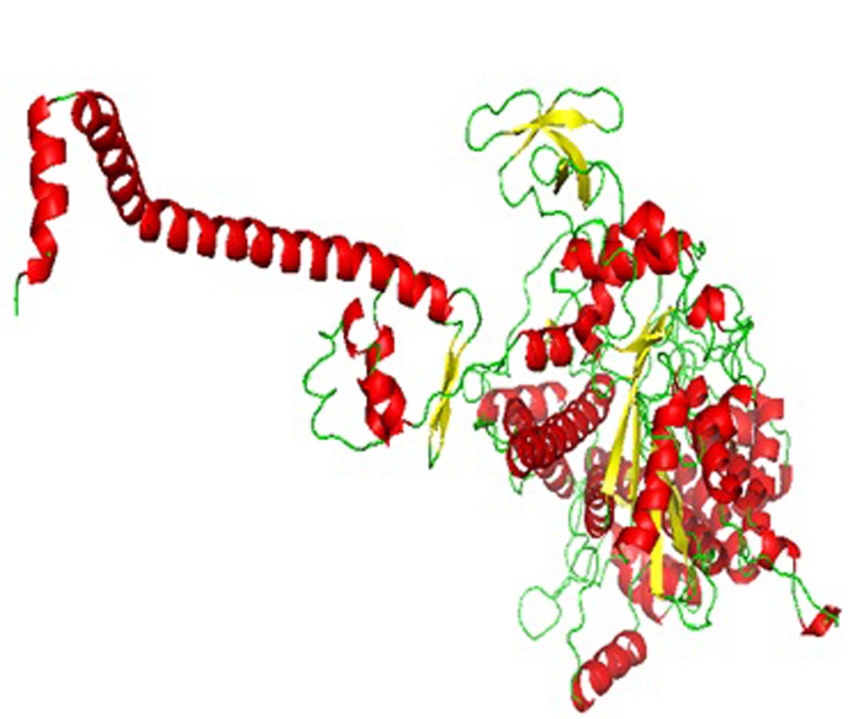


**Figure S4 The 3D homologous model of MYH1.**The tertiary structure of native MYH1is drawn here as a cartoon model.

**
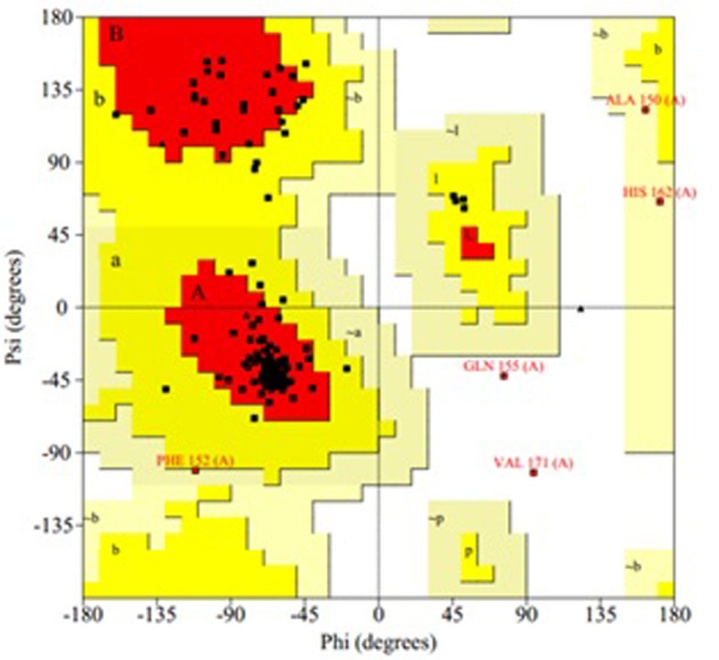
**

**Figure S5 Ramachandran Plot of native MYH1**. The red, brown, and yellow regions represent the favored, allowed, and "generously allowed" regions as defined by ProCheck for MYH1.

.

**
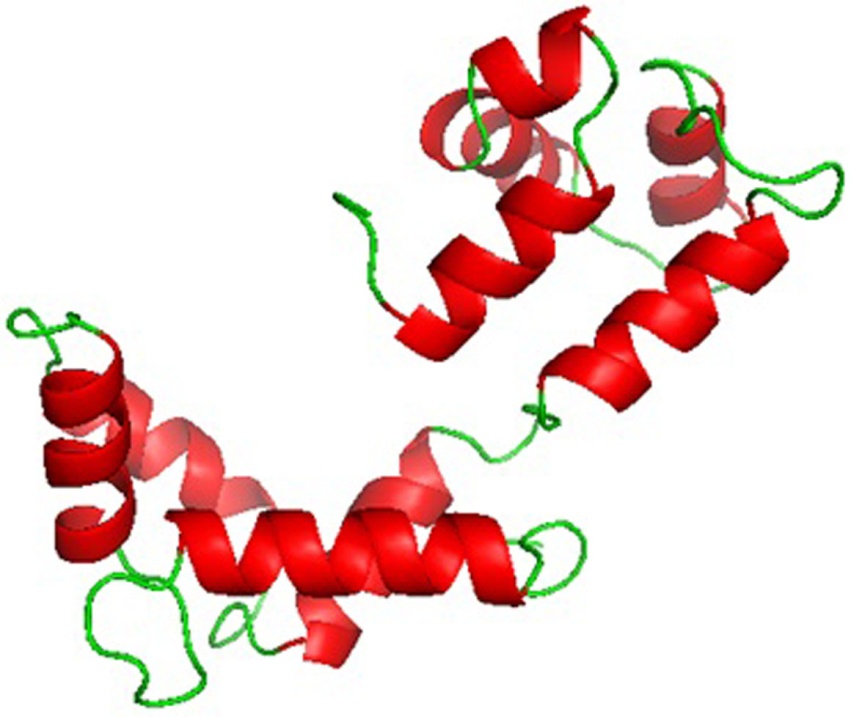
**

**Figure S6 The 3D homologous model of MYLPF**. The tertiary structure of MYLPF is drawn here as a cartoon model.


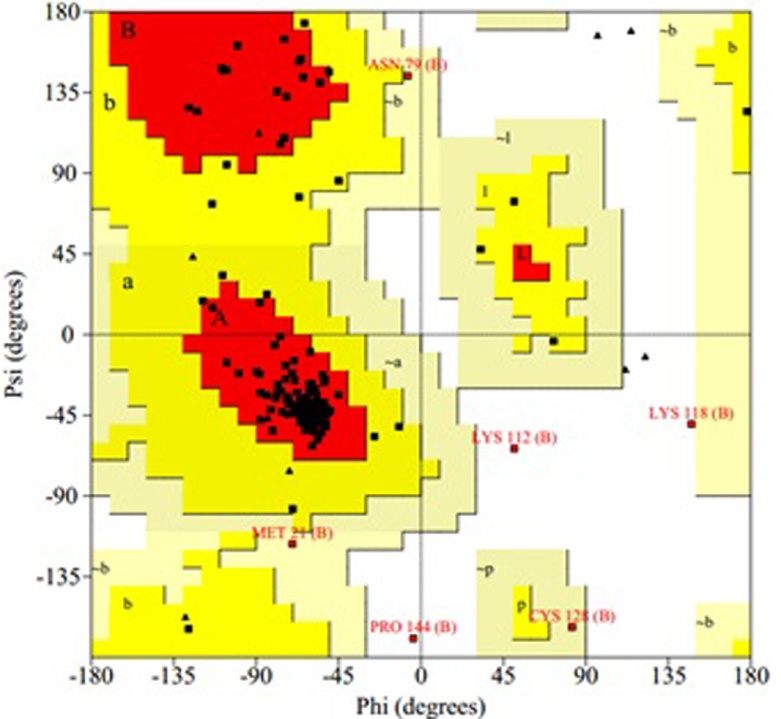


**Figure S7 Ramachandran Plot of MYLPF**. The plot represents the favored, allowed, and "generously allowed" regions as defined by ProCheck for MYLPF.


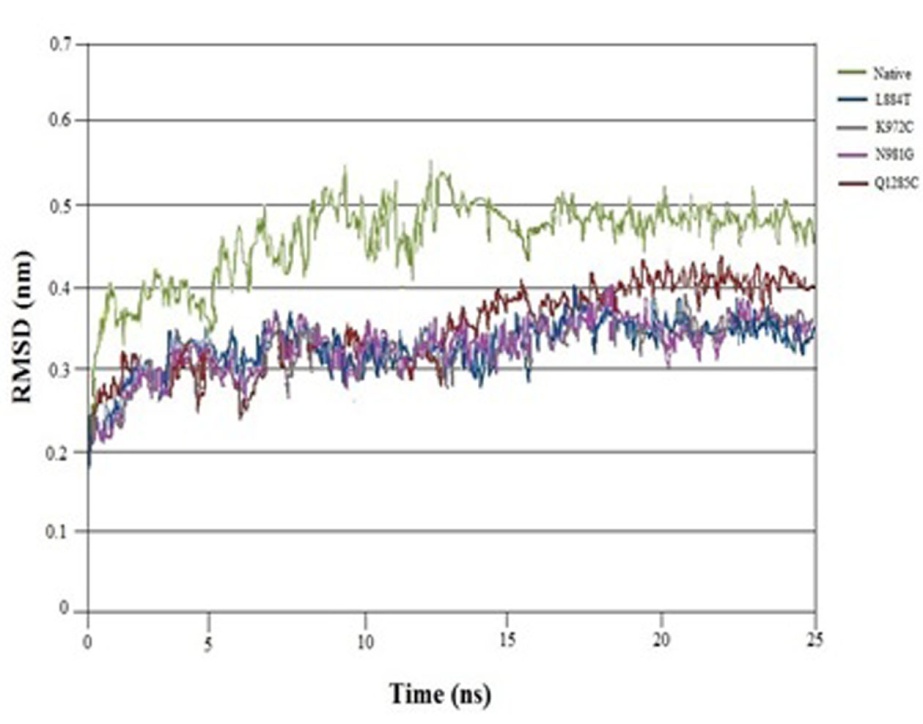


**Figure S8** **The RMSD values of all backbone atoms of MYH1- MYLPF protein complexes.** The RMSD (in nanometer) is at ordinate and Time (in nano-second) at abscissa. The structural simulations for the native *MYH1* (green), mutant *MYH1* L884T (blue), K972C (black), N981G (pink) and Q1285C (red) complexes with MYLPF.
