# Supplementary material for: An integrated in silico approach for functional and structural impact of non- synonymous SNPs in the MYH1 gene in Jeju Native Pigs
Source: BMC Genet. 2016 Feb 4;17:35. doi: 10.1186/s12863-016-0341-1 (PMC4741023; doi:10.1186/s12863-016-0341-1)
Supplement: Additional file 2: — The ARRIVE Guidelines Checklist Animal Research: Reporting In Vivo Experiments. (PDF 386 kb) [file 12863_2016_341_MOESM2_ESM.pdf]

ARRIVE

The ARRIVE Guidelines Checklist
Animal Research: Reporting In Vivo Experiments

	ITEM	RECOMMENDATION	Section/Paragraph
Title	1	Provide as accurate and concise a description of the content of the article as possible.	An integrated in silico approach for functional and structural impact of non-synonymous SNPs in the MYH1 gene in Jeju Native Pigs (Page:1)
Abstract	2	Provide an accurate summary of the background, research objectives, including details of the species or strain of animal used, key methods, principal findings and conclusions of the study.	BACKGROUND<br>This study was performed to identify the non- synonymous polymorphisms in the myosin heavy chain 1 gene (MYH1) association with skeletal muscle development in economically important Jeju Native Pig (JNP) and Berkshire breeds. (Page:3)<br><br>RESULTS<br>The NextGENe (V 2.3.4.) tool was used to identify the variants in MYH1 from JNP and Berkshire using RNA seq. The 95% confidence intervals clearly indicate that the mRNA expression of MYH1 is significantly higher in the Berkshire *longissimus dorsi* muscle samples than JNP breed. Concordant in silico analysis of MYH1, the open-source software tools identified 4 potential nsSNP (L884T, K972C, N981G, and Q1285C) in JNP and 1 nsSNP (H973G) in Berkshire pigs. The results of molecular docking studies on MYH1 (native and 4 mutants) and MYLFP demonstrated that the native complex showed higher electrostatic energy (-466.5 Kcal mol-1), van der Walls energy (-87.3 Kcal mol-1), and interaction energy (-835.7 Kcal mol-1) than the mutant complexes. The molecular dynamic simulation revealed that the native complex yielded a higher root-mean-square deviation (0.2–0.55 nm) and lower root-mean-square fluctuation (approximately 0.08–0.3 nm) as compared to the mutant complexes. (Page: 3)<br><br>CONCLUSIONS<br>The results suggest that the variants at L884T, K972C, N981G, and Q1285C in MYH1 in JNP might represent a cause for the poor growth performance for this breed. This study is a pioneering in-depth in silico analysis of polymorphic MYH1 and will serve as a valuable resource for further targeted molecular diagnosis and population-based studies conducted for improving the growth performance of JNP. (Page: 4)

**INTRODUCTION**			
Background	3	a. Include sufficient scientific background (including relevant references to previous work) to understand the motivation and context for the study, and explain the experimental approach and rationale.	Skeletal muscle genes are potential candidate genes that can functionally influence livestock production and meat quality. Research on the relationships between skeletal muscle characteristics and meat quality is crucial for improving our understanding of the molecular basis of skeletal muscle phenotypes (Karlsson et al., 1999). Myosin is the most abundant protein expressed in striated muscle cells: myosin makes up ~25% of the total protein pool and its isoforms are mainly expressed in skeletal muscle during different developmental stages, including the embryonic period, and therefore play a role in the development of skeletal muscle (Lijun et al., 2013). Pigs are a key source of meat and are widely consumed in several countries. During the last decade, pork meat quality has been targeted in large breeding programs, and has therefore been the focus of a substantial amount of research. Swine has attracted attention as a valuable non-rodent animal model for both livestock and biomedical researches (Swindle et al., 2012). (Page: 5-6)<br><br>For this purpose, we selected Jeju Native Pig (JNP) and Berkshire. JNP an indigenous breed of swine that is found at Jeju-Do, is particularly desired by consumers because its meat is delicious. However, low feed efficiency, small litter size and small adult body weight are major drawbacks. Berkshire breed gains weight more efficiently and yields leaner meat as compared to Landrace and Western breeds. Moreover, it's closely related to Asian native pigs. (Page: 6)
		b. Explain how and why the animal species and model being used can address the scientific objectives and, where appropriate, the study's relevance to human biology	Standard farm pigs have been used with increasing frequency in a variety of research areas including metabolic, liver, reproduction, cardiovascular, toxicology, pharmacology, pulmonology and infection disease. Berkshire and Korean native pig breeds renowned for its palatability characteristics are also been used in translational bioresearch (Jung et al., 2012)
Objectives	4	Clearly describe the primary and any secondary objectives of the study, or specific hypotheses being tested.	Herein, we present an in silico analysis, with a focus on (a) in silico approaches to predict the functional effect of non-synonymous SNP (nsSNP) in MYH1 on growth, and (b) molecular docking and dynamic simulation of MYH1 to predict the effects of those nsSNP on protein-protein association. Further, quantitative trait loci (QTL) are biologically meaningful loci at which gene expression is modified by genotype. (Page: 7)
**METHODS**			
Ethical statement	5	Indicate the nature of the ethical review permissions, relevant licences (e.g. Animal [Scientific Procedures]	This study was conducted under strict accordance with the recommendations in the guide for the care and use of animals of the

		Act 1986), and national or institutional guidelines for the care and use of animals, that cover the research.	Animal Bioethics Committee (permit number: 2013-0009) of Jeju National University, Jeju-Si, Jeju-Do, Republic of Korea. The animals were handled accordance with proper animal welfare guidelines (Garber JC, 2011) (Page: 22)
Study design	6	For each experiment, give brief details of the study design including:<br><br>a. The number of experimental and control groups.<br>b. Any steps taken to minimise the effects of subjective bias when allocating animals to treatment (e.g. randomisation procedure) and when assessing results (e.g. if done, describe who was blinded and when).<br>c. The experimental unit (e.g. a single animal, group or cage of animals).<br><br>A time - line diagram or flow chart can be useful to illustrate how complex study designs were carried out.	Present study included a total numbers of 10 animals (n=5 from each breed). The longissimus dorsi muscle samples were collected from 8 months full growth adult pigs, considered as the economical tread time period in South Korea when the farmer sold out the livestock for slaughter. The numbers for the experiment animals were decided based on the minimum average relatedness and according to method described by Erb (1990) and Charan (2013). (Page: 23)<br><br>All the pigs were provided ad libitum access to commercial feed (Seoul Feed, Jeju-Si, South Korea) and water. Pigs were housed in concrete-floored pens that contained a nipple-bowl drinker and a feeder. Animals were sacrificed through entailed exsanguination following electric stunning, with all possible effort being devoted to minimize suffering. (Page: 22-23)<br><br>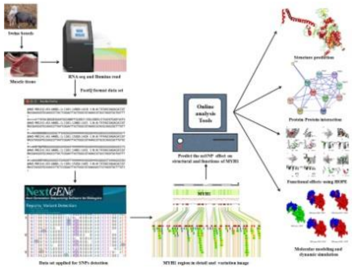 Figure 1 The schema of the semantics *in silico* analysis of the structural and functional assessment of MYH1. *In silico* methods were used carefully to evaluate the ontology of *MYH1* gene function, protein association network for MYH1 and the effects of the nsNSPs on the MYH1 functions. (Page: 24)
Experimental procedures	7	For each experiment and each experimental group, including controls, provide precise details of all procedures carried out. For example:<br><br>a. How (e.g. drug formulation and dose, site and route of administration, anaesthesia and analgesia used [including monitoring], surgical procedure,	Animals were sacrificed through entailed exsanguination following electric stunning, with all possible effort being devoted to minimize suffering. (Page: 22)

		method of euthanasia). Provide details of any specialist equipment used, including supplier(s).<br><br>b. When (e.g. time of day).<br><br>c. Where (e.g. home cage, laboratory, water maze).<br><br>d. Why (e.g. rationale for choice of specific anaesthetic, route of administration, drug dose used).	 <br><br>All other experiments were conducted in the light phase
Experimental animals	8	a. Provide details of the animals used, including species, strain, sex, developmental stage (e.g. mean or median age plus age range) and weight (e.g. mean or median weight plus weight range).<br><br>b. Provide further relevant information such as the source of animals, international strain nomenclature, genetic modification status (e.g. knock - out or transgenic), genotype, health/immune status, drug or test naive, previous procedures, etc.	Pure-breed adult female animals from JNP and Berkshire breeds (n=5 from each breed; average individual body weight, 84.76±3.5 kg) (Page: 22)<br><br>The piglets of the breeds were collected from the Jeju Province Livestock preservation organization and the health reports indicated that the rats were free of known viral, bacterial and parasitic pathogens.
Housing and husbandry	9	Provide details of:<br>a. Housing (type of facility e.g. specific pathogen free [SPF]; type of cage or housing; bedding material; number of cage companions; tank shape and material etc. for fish).<br><br>b. Husbandry conditions (e.g. breeding programme, light/dark cycle, temperature, quality of water etc for fish, type of food, access to food and water, environmental enrichment).<br><br>c. Welfare-related assessments and interventions that were carried out prior to, during, or after the experiment.	Pigs were housed in concrete-floored pens that contained a nipple-bowl drinker and a feeder. (Page: 22)<br><br>Piglets were weaned immediately after birth and moved to a common nursery maintained at an ambient temperature 25 ± 1 °C. All the pigs were provided *ad libitum* access to commercial feed (Seoul Feed, Jeju-Si, South Korea) and water.
Sample Size	10	a. Specify the total number of animals used in each experiment, and the number of animals in each experimental group.<br><br>b. Explain how the number of animals was arrived	Each group consisted with five animals. The breeds were reared under the same environmental and nutritional conditions till 8 months till the weight considered as 84.76±3.5 kg in this study. (Page: 22)<br><br>The numbers for the experiment animals were decided based on the

		at. Provide details of any sample size calculation used.<br><br>c. Indicate the number of independent replications of each experiment, if relevant.	minimum average relatedness and according to method described by Erb (1990) and Charan (2013). (Page: 15)<br><br>The experiments were repeated in triplicate, and data were pooled. (Page: 31)
Allocating animals to experimental groups	11	a. Give full details of how animals were allocated to experimental groups, including randomisation or matching if done.<br>b. Describe the order in which the animals in the different experimental groups were treated and assessed.	For experiments using animals, the pigs were ranked in ascending order and marked with ear tag i.e., J1 to J5 for JNP breed and B1 to B5 for Berkshire.
Experimental outcomes	12	Clearly define the primary and secondary experimental outcomes assessed (e.g. cell death, molecular markers, behavioural changes).	Two primary outcome measures were analyzed, Both RT-PCR and real-time qRT-PCR were performed to clarify the qualitative and quantitative expressions of the gene under study and In addition, the protein expression for the MYH1 obtained by western blot has also been presented as relative band intensities between the breeds. In addition in silico approaches to predict the functional effect of non-synonymous SNP (nsSNP) in MYH1 on growth, and (b) molecular docking and dynamic simulation of MYH1 to predict the effects of those nsSNP on protein-protein association. Further, the QTL map in *Sus scrofa* chromosome 12 from recently published studies as a means to assess the integrity of sequencing that can support this study about the biologically meaningful loci at which gene expression. (Page: 7)
Statistical methods	13	a. Provide details of the statistical methods used for each analysis.<br><br>b. Specify the unit of analysis for each dataset (e.g. single animal, group of animals, single neuron).<br><br>c. Describe any methods used to assess whether the data met the assumptions of the statistical approach.	Haplotype blocks and linkage disequilibrium (LD) plots have been constructed using Haploview version 4.2 with the default algorithm. After removing outliers (Grubb's test) by dividing the covariance of the data sets with the product of their standard deviations the Pearson's correlation coefficient (*r*) was calculated. (Page: 31)<br><br>The relative quantitative (RQ) of *MYH1* expression was studied by means ± SEM of 5 animals with triplicates ($P<0.05$). To correct for technical inter-run variation among triplicate reactions of the same sample measured in different runs, the data were calibrated by calculating the average cycle threshold (Ct) value over all the samples in each run. After the calibration, the average Ct-value of each triplicate reaction was converted to relative quantities and these were analyzed using Tukey's b test. (Page: 31)
**RESULTS**			
Baseline data	14	For each experimental group, report relevant characteristics and health status of animals (e.g.	The animals' health status was monitored throughout the experiments by a health surveillance programme according to Federation of European

<table>
<tr><td></td><td></td><td>weight, microbiological status, and drug or test naïve) prior to treatment or testing. (This information can often be tabulated).</td><td>Laboratory Animal Science Associations (FELASA) guidelines. The animals were free of all viral, bacterial, and parasitic pathogens listed in the FELASA recommendations</td></tr>
<tr><td>Numbers analysed</td><td>15</td><td>a. Report the number of animals in each group included in each analysis. Report absolute numbers (e.g. 10/20, not 50%[2]).

b. If any animals or data were not included in the analysis, explain why.</td><td>All animals from each of the breed are included in the data analysis.</td></tr>
<tr><td>Outcomes and estimation</td><td>16</td><td>Report the results for each analysis carried out, with a measure of precision (e.g. standard error or confidence interval).</td><td>In accordance with the ARRIVE guidelines (Kilkenny et al. 2010), we have reported measures of precision, confidence, and n to provide an indication of significance.

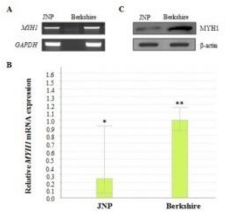

Figure 2 Expression analysis of mRNA of MYH1 in JNP and Berkshire. (A) Expression of MYH1 mRNA in 1% agarose gel at RT-PCR. (B) Relative quantitative has shown the significant expression differences of MYH1 gene between two breeds. Error bars represent the 95% confidence interval. (C) Relative differential blot expression analysis of MYH1 proteins in JNP and Berkshire. (Page: 8-9)</td></tr>
<tr><td>Adverse events</td><td>17</td><td>a. Give details of all important adverse events in each experimental group.

b. Describe any modifications to the experimental protocols made to reduce adverse events.</td><td>The animals' health status was regularly monitored and make sure to keep animals free of all viral, bacterial, and parasitic pathogens</td></tr>
<tr><td colspan="4">DISCUSSION</td></tr>
<tr><td>Interpretation/ scientific implications</td><td>18</td><td>a. Interpret the results, taking into account the study objectives and hypotheses, current theory and other relevant studies in the literature.</td><td>This study represents the first comprehensive investigation that has identified functional nsSNP in MYH1in JNP and Berkshire breeds by using sequence- and structure-based homology algorithms. In silico annotation of certain nsSNP could explain the functional effects of these mutations. Furthermore, pathway-based analysis of protein-protein interactions highlighted the importance of the interaction between MYH1 and MYLPF in skeletal muscle development. The results of this study suggest that the variants L884T, K972C, N981G, and Q1285C in MYH1 in JNP might</td></tr>
</table>

			represent a cause for the poor growth performance of this breed. Thus, these MYH1 variants might be useful as selection markers for improving growth performance in the JNP breed. (Page: 22)
		b. Comment on the study limitations including any potential sources of bias, any limitations of the animal model, and the imprecision associated with the results[2].	Further, study is need for performance test to confirm the associations within and between the breeds and the degree of correlation among the large sample size that will guide us to include the identified nsSNP for breed improvement program.
		c. Describe any implications of your experimental methods or findings for the replacement, refinement or reduction (the 3Rs) of the use of animals in research.	The sample size and have demonstrated that selection based on minimum average relatedness contributes to high percentage of polymorphic markers and are also indicative of a signature for homozygosity for each breed. The selected nsSNPs in this study have been chosen as marker due to their high stability, density and the highly automated way in which SNPs assays are performed and detected. Therefore the less number of animals and throughput analysis can also be representative to state the specificity of the SNP in a breed for the population.
Generalisability/ translation	19	Comment on whether, and how, the findings of this study are likely to translate to other species or systems, including any relevance to human biology.	The identification of the *MYH1* gene and their mutants suggested that *MYH1* play a potential role in porcine body growth. In addition QTL mapping of *MYH1* also supported the *in silico* evidence about the association with the meat quality and marbling. Thus it is propose that *MYH1* gene can be used as candidate marker for the meat quality and livestock improvement program. (Page: 21)
Funding	20	List all funding sources (including grant number) and the role of the funder(s) in the study.	This study was supported by a grant from the Next-Generation BioGreen 21 Program (No. PJ01117401), Rural Development Administration, Republic of Korea, hence the authors are thankful to this organization. (Page: 35)

References:

Karlsson AH, Klont RE, Fernandez X. Skeletal muscle fibers as factors for pork quality. Livest Prod Sci. 1999; 60: 255–269.

Lijun W, Xiaolin L, Fubiao N, Hongliang W, Hua H, Yulan G. Single nucleotide polymorphisms, haplotypes and combined genotypes in MYH3 gene and their associations with growth and carcass traits in Qinchuan cattle. Mole Biol Rep. 2013; 40: 417-426.

Swindle MM, Makin A, Herron AJ, Clubb Jr FJ, Frazier KS. Swine as models in biomedical research and toxicology testing. Vet Pathol. 2012; 49(2): 344-356.

Jung WY, Yu Sl, Seo DW, Jung KC, Cho IC, Lim HT, Jin DI, Lee JH. 2012. Characterization of insertional variation of Procine endogenous retroviruses in six differrernt pig breeds. Asian-Aust. J. Anim. Sci. 25(10) : 1357 – 1363.

Garber JC. Guide for the Care and Use of Laboratory Animals. 8th ed. The National Academies Press: Washington, DC; 2011

Erb HN. A statistical approach for calculating the minimum number of animals needed in research. ILAR J. 1990; 32 (1): 11-16.

Charan J, Kantharia ND. How to calculate sample size in animal studies? J Pharmacol Pharmacother. 2013; 4(4): 303–306.

Kilkenny C, Browne WJ, Cuthill IC, Emerson M, Altman DG. Improving Bioscience Research Reporting: The ARRIVE Guidelines for Reporting Animal Research. PLoS Biol. 2010; 8(6): e1000412. doi:10.1371/journal.pbio.1000412
